# Shell-Nested Atomic Orbitals: Restoring the Correlation-Consistent Hierarchy inside the Molecular Virtual Space

**DOI:** 10.1021/acs.jpca.6c06093

**Published:** 2026-10-20

**Authors:** Dariusz W. Szczepanik

**Affiliations:** K. Gumiński Department of Theoretical Chemistry, Faculty of Chemistry, 37799 Jagiellonian University, Gronostajowa 2, Kraków 30-387, Poland

## Abstract

Correlation-consistent basis sets enlarge the one-electron space atom by atom and shell by shell, but the canonical molecular virtual space retains no comparable atom-resolved hierarchy. Extending the frozen natural-minimal-basis (NMB) construction, we define shell-nested atomic orbitals (SNAOs): each cycle selects the highest-occupation residual radial function from every eligible atomic angular-momentum channel, assembles the selected functions into a complete layer, and freezes that layer. The levels *L*_1_ ⊂ *L*_2_ ⊂···share one parent-basis restricted Hartree–Fock reference; only the virtual degrees of freedom available to dynamic correlation are increased. The level index therefore provides a common coordinate for examining radial and angular saturation. Benchmarks comprise five hydrides and Ne through cc-pV7Z, benzene, pyrrole, and furan, and 32 closed-shell first-row molecules in cc-pV5Z. Rank-matched frozen natural orbitals (FNOs) recover more correlation energy at each finite dimension, as expected for an occupation-optimized compressor. The SNAO sequence is less compact but more regular: for the 32-molecule set, the three-point *L*_2_–*L*_4_ limit differs from the two-point cc-pV­(Q,5)­Z estimate by 3.2 m*E*_*h*_ on average, compared with 13.1 m*E*_*h*_ for FNO. SNAO thus complements FNO by exchanging some fixed-rank recovery for a transferable, shell-resolved sequence suited to the analysis and extrapolation of correlation freedom.

## Graphical abstract

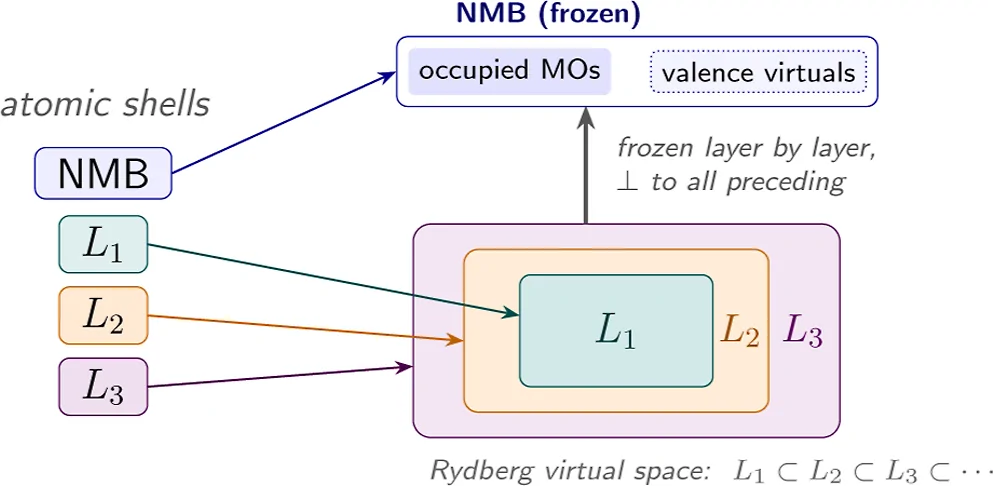

## Introduction

Correlation-consistent basis sets provide a systematic route toward the complete-basis-set (CBS) limit by enlarging the one-electron space atom by atom and shell by shell. Along an ordinary basis-set sequence, however, the optimized occupied Hartree–Fock space and the virtual manifold change together. The present work introduces a complementary construction in which one parent-basis restricted Hartree–Fock (RHF) reference is held fixed while only the virtual freedom available to dynamic correlation is increased. The resulting levels therefore provide a common reference for asking how radial and angular correlation degrees of freedom become saturated.

This question follows directly from the frozen natural-minimal-basis (NMB) construction. The natural atomic orbital (NAO) method of Reed, Weinstock, and Weinhold^1–3^ is a mature, density-based route to atom-centered orbitals and populations. In our preceding study,^4^ we showed that the AO expansions of valence NAOs can acquire large, mutually canceling contributions near the CBS limit. Frozen-NMB avoids this sensitivity by defining and freezing the chemically dominant core-plus-valence space before rebuilding the full Rydberg complement. Once the NMB has been secured, however, the remaining Rydberg space is still formed in one global cycle. The present work asks whether the same freezing principle can organize that space recursively, one complete atomic layer at a time.

Most virtual-space truncation methods rank orbitals by their importance for a particular correlated wave function. Frozen natural orbitals (FNOs)^5–7^ and pair natural orbitals^8,9^ use molecular natural-orbital occupations, whereas concentric localization^10^ generates successive virtual shells from singular-value decompositions. These methods are designed to recover as much correlation energy as possible at a chosen dimension. Their orderings are intentionally molecular and threshold-based, and need not define the same atom- and shell-labeled subspace when the rank, molecule, or parent basis changes.

Atomic natural orbitals^11^ and Dunning’s correlation-consistent basis sets^12,13^ suggest a different organizing coordinate: atom-centered radial functions are added in complete angular-momentum layers. This shell schedule is a pragmatic basis-set design principle, not an assertion that all members of a layer contribute identical correlation energy. Deviations are expected for deep layers and outside the light-element regime examined here. What remains useful is the ordered, chemically labeled sequence itself. A molecular hierarchy that preserves this sequence relative to one fixed occupied reference can separate radial and angular saturation from changes in the Hartree–Fock solution.

We construct such a hierarchy by extending frozen-NMB recursively. The first cycle is exactly the frozen-NMB construction. Each subsequent cycle selects the highest-occupation residual radial function from every eligible atomic angular-momentum channel, combines the selected functions into a complete layer, orthonormalizes that layer, and freezes it. The accumulated shell-nested atomic orbitals (SNAOs) define *L*_1_ ⊂ *L*_2_ ⊂···throughout the virtual space. At a matched rank, FNO is expected to recover more correlation energy because it is optimized directly for the supplied correlated density. The difference between the FNO and SNAO energies therefore measures the finite-space cost of imposing an atomically resolved, nested shell order. The benefit of that order is a regular sequence rather than an optimal isolated point: successive levels can be compared across parent bases and used as an internal extrapolation coordinate.

The same hierarchy also creates a route to domain-resolved analysis. After localizing the occupied space, the incremental correlation recovered upon adding each SNAO layer can in principle be assigned to individual lone pairs, σ or π bonds, orbital pairs, or fragments. Different bonding motifs need not exhaust their correlation freedom at the same shell depth. The present benchmarks focus on the global hierarchy and its extrapolation, but the atom- and shell-resolved construction already supplies the common coordinate required for such local comparisons.

We test the hierarchy in four respects: its effect on natural populations, the radial order of its shell occupations, the identity of its subspaces across a cc-pV*n*Z sequence, and the convergence of correlation energies in level-truncated spaces. The benchmarks comprise five hydrides and Ne in bases through cc-pV7Z, three aromatic rings, and 32 closed-shell first-row molecules in a cc-pV5Z parent. The central distinction is between fixed-rank compression and convergence of a sequence: FNO gives the better finite subspace, whereas SNAO is designed to provide the more regular, transferable path through virtual-space dimension.

## Theory

### Notation and the Frozen-NMB Construction

Let **S** be the AO overlap matrix and **D** the spinless one-electron density matrix in the AO basis. The occupation of an **S**-normalized vector **v** of AO coefficients is the Rayleigh quotient

1 $$n_{v}=v^{T}Mv,,M=SDS$$

A channel (*A*, *l*) denotes the set of radial basis functions of angular momentum *l* on atom *A*; every per-channel eigenproblem below is solved on matrices averaged over the 2*L* + 1 magnetic components, and the resulting radial rotation is replicated identically over all *m*, which keeps the construction covariant under molecular rotations.^1^

The frozen-NMB construction^4^ separates two operations that are simultaneous in the standard full-space NAO driver. The first is to define a compact, density-adapted core-plus-valence NMB in a well-conditioned per-atom space; the second is to complete that fixed NMB with an orthogonal Rydberg complement spanning the remainder of the AO space. The underlying idea of a distinguished, molecule-adapted minimal atomic set goes back to reference-minimal-basis population analysis.^14,15^

Cycle 1 runs the standard NAO algorithm in the conditioned basis and lifts the resulting core and valence NAOs to the full basis, where they are frozen without further rotation. Cycle 2 then reconstructs the complete Rydberg complement in one global occupation-weighted symmetric orthogonalization (OWSO). This ordering is sufficient when the objective is a stable NMB and a complete NAO transformation, but it does not impose a radial hierarchy on the Rydberg orbitals. For self-consistent-field (SCF) densities such an order often emerges because the Rydberg occupations fall rapidly. Correlated densities have a flatter distribution of Rydberg occupations, and the final global orthogonalization can redistribute occupation between neighboring radial directions, as examined below in the radial-occupation analysis.

### The Shell-Nested Construction

The shell-nested construction (Figure 1) replaces the single Rydberg cycle by a sequence in which one shell layer is selected and frozen at a time. Cycle 1 and the resulting NMB are identical to the frozen-NMB construction. The remaining AO space is maintained **S**-orthogonal to every previously frozen layer. Cycle *k* = 1, 2, ... then acts on each channel (*A*, *l*) that satisfies the eligibility rule

2 $$l\leq l_{val}\left(A\right)+k$$

­(with *l*_val_ the highest angular momentum of the occupied valence shells of the free atom), the following steps.

1. canonically orthonormalize the residual radial functions of the channel, removing directions already represented in the frozen space and any numerical linear dependencies;
2. Diagonalize the *m*-averaged occupation form, eq 1, and retain the eigenvector with the largest eigenvalue as the natural radial function of the current layer;
3. Collect the selected vectors into one shell layer, project that layer once more against the full frozen space, orthonormalize it internally by OWSO followed by a final symmetric orthonormalization,^16^ and freeze it.

A complete matrix formulation of these steps is given in the Supporting Information. Later cycles leave previously frozen orbitals unchanged. The shell schedule shown in Figure 1b follows the bookkeeping of the correlation-consistent hierarchy:^12^ each cardinal introduces the next angular-momentum channel together with further radial flexibility. The construction uses this ordered shell content as a coordinate; it does not assume that all functions introduced at one level recover identical amounts of correlation energy. A channel can contribute only if it is present in the computational basis, so an early layer may be incomplete in a small basis; the recursion ends when all radial directions have been selected. Orbitals are labeled *n*(*l*) = *l* + 1 + *u*, where *u* is the number of radial functions already selected from that channel, and the principal label therefore increases with the layer index.

**Figure 1:**
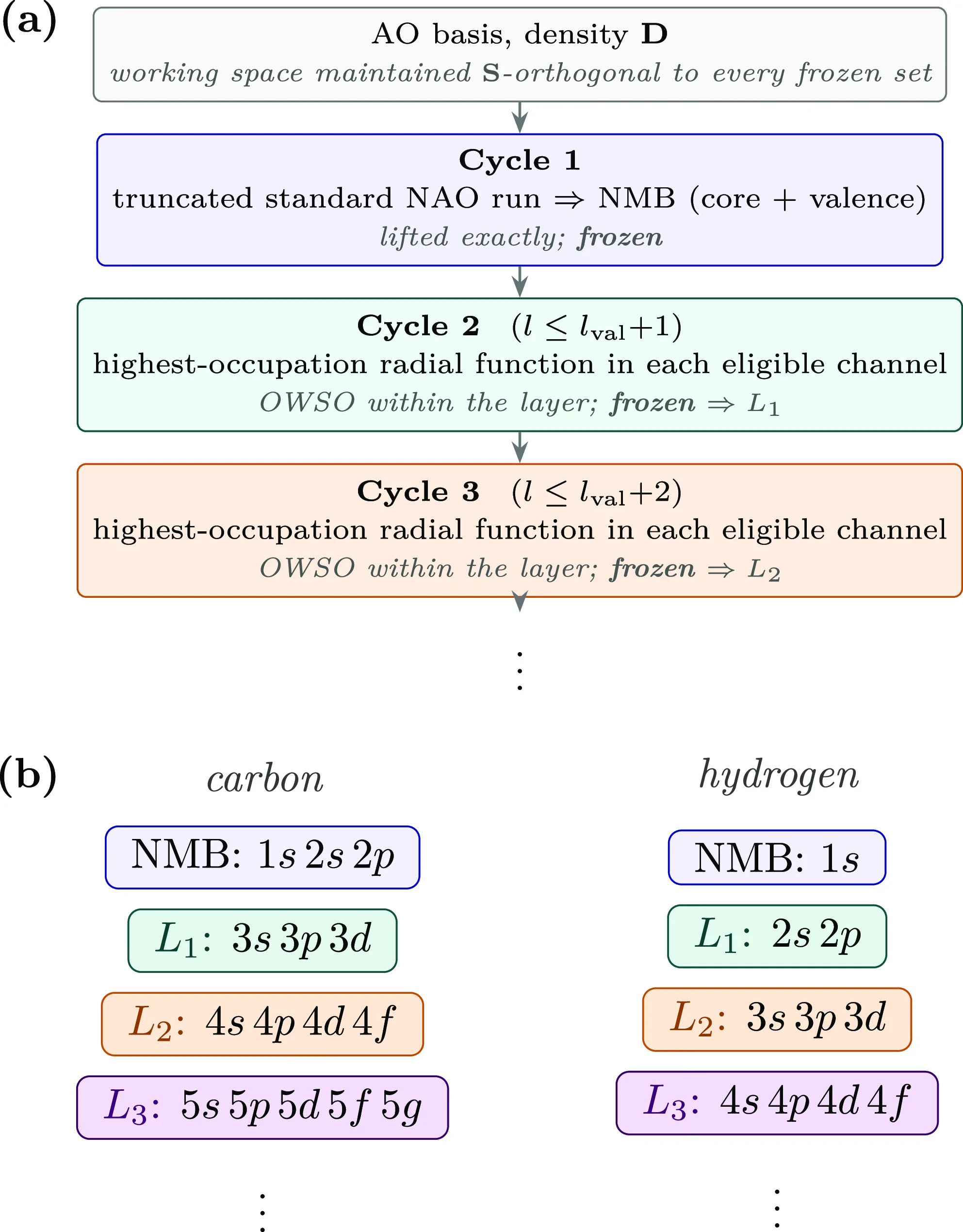
(a) The shell-nested construction. Cycle 1 defines and freezes the NMB exactly as in the frozen-NMB construction; each subsequent cycle selects the highest-occupation residual radial function from every eligible channel, orthonormalizes the selected functions as one shell layer, and freezes that layer. Every subsequent selection is made in the orthogonal complement of all preceding layers, so the accumulated spaces are nested by construction. (b) The resulting shell progression for methane: each correlation layer carries the color of the cycle that freezes it, and the pattern continues through the deeper layers.

### Formal Properties

The construction has five useful properties; a proof of property (ii) is given in the Supporting Information.

(i) NMB identity*.* Cycle 1 is exactly the frozen-NMB procedure, and later cycles leave its columns unchanged. The NMB is therefore numerically identical to the frozen-NMB result.
(ii) Monotonic radial selection. The admissible radial space of a channel can only shrink from layer *k* to layer *k* + 1. Its maximum Rayleigh quotient therefore cannot increase, so the occupations selected from each channel form a nonincreasing sequence for either an SCF or a correlated density. Within-layer OWSO may subsequently redistribute a small amount of occupation among overlapping functions on different atoms; in the present benchmarks such changes are confined to nearly unoccupied channels, as shown in the subsection “Radial occupations recover shell order.” For an isolated atom there is no interatomic overlap and the ordering remains exact.
(iii) Rotational covariance and Pauli bounds. All channel selections are *m*-averaged, and every occupation is a Rayleigh quotient of (**D**, **S**). The resulting occupations therefore remain within the eigenvalue range of the input density.
(iv) Completeness. Apart from numerical linear dependencies, the final transformation spans the full AO space and is **S**-orthonormal to numerical precision.
(v) Nested shell content. The layer index fixes which atomic angular channels are eligible, while a larger member of the same systematic basis family refines those channels and supplies any missing higher-angular-momentum functions. The shell composition is therefore stable by construction. Whether the corresponding molecular subspaces converge to the same physical spaces is a separate quantitative question addressed in the cross-basis nesting analysis below. The algebraic recursion can be applied to any atom-centered parent basis, but identifying the level index with a basis-set cardinal requires a family with a systematic shell progression. That interpretation is tested here only for first-row cc-pV*n*Z spaces.

### Relation to Existing Constructions

The channel eigenproblem in step 2 is an atomic-natural-orbital construction^11^ carried out in the molecular environment, on the *m*-averaged atomic density block and under a frozen-subspace constraint. The level index then plays a role analogous to an occupation threshold in frozen-natural-orbital truncation:^6,7^ it determines the size of the retained space, but it does so through a fixed atom-and-shell composition rather than a molecule-dependent cutoff.

The distinction is one of purpose. FNOs identify the molecular virtual directions that carry the largest share of a correlated density and are therefore designed for efficient fixed-rank compression. SNAOs impose an additional constraint: the retained spaces must form a nested sequence of complete atom-centered shell layers. At a matched dimension, the difference between the FNO and SNAO correlation energies is therefore the energetic price of this organization. What is gained is a level index whose chemical meaning does not depend on a global occupation threshold and whose increments can be compared from one system or parent basis to another.

This comparison is especially clean because every truncated calculation retains the same occupied RHF orbitals of the full parent basis. Conventional CBS sequences change both the optimized reference and the available virtual space. The SNAO sequence changes only the latter and therefore isolates the radial and angular freedom admitted to dynamic correlation. It can be used not only as a global extrapolation coordinate but also, after localization of the occupied space, as a common coordinate for domain-resolved convergence. The increments associated with a lone pair, a σ or π bond, an occupied pair, or a fragment can then be followed layer by layer. Such domain-resolved applications are not evaluated numerically here, but they are a direct consequence of the labeled nested spaces defined by the present construction.

The standard NAO construction^1,3^ orthogonalizes the full Rydberg space in one global step; frozen-NMB^4^ freezes the NMB before that step; the shell-nested construction extends the freeze to each successive shell layer. The construction itself is independent of how the input density was obtained. The present use of an MP2 density is a controlled way to compare SNAO and FNO with identical correlated information, not a claim that generating an MP2 density is a cost-free production route.

### Computational Methods

All NAO constructions were performed with runsnvo,^17^ which implements the standard (NBO7-equivalent), frozen-NMB,^4^ and shell-nested algorithms in one program and writes the level-truncated virtual spaces used in the correlation-energy analyses below (the shell-nested virtual orbitals, SNVOs, of the program name). The shell-nested procedure is selected with –nao-algorithm snao. All comparisons therefore start from identical density matrices and differ only in the NAO construction.

Three benchmark sets were used. The first comprises the five hydrides BH_3_, CH_4_, NH_3_, H_2_O, and HF, together with the Ne atom, at fixed experimental geometries^18^ listed in the Supporting Information. Restricted Hartree–Fock (RHF) and unrelaxed coupled-cluster singles and doubles (CCSD) densities were obtained with ORCA 6.1.1 ^19^ in the cc-pV*n*Z basis sets,^12,20^ *n* = D–7. The second set contains benzene, pyrrole, and furan at the B3LYP-D3BJ/cc-pV5Z geometries of ref 4, with B3LYP-D3BJ densities from Gaussian 16 ^21^ over the same basis-set sequence. A single fixed geometry was used along each sequence, so the subspaces obtained at different cardinals can be compared directly. The third set comprises 32 closed-shell molecules of H, C, N, O, and F with one to three heavy atoms, from F_2_ and N_2_ to ethanol and cyclopropane (Figure 7). Starting structures were taken from PubChem3D^22^ (F_2_ and allene were built from experimental parameters) and optimized at the r^2^SCAN-3c level^23^ with ORCA 6.1.1; all structures are verified minima, and their coordinates are listed in the Supporting Information.

The cross-basis nesting analysis compares subspaces obtained for one molecule in two basis sets, *a* and *b*. Let **C**_*a*_ and **C**_*b*_ be the **S**-orthonormal AO-coefficient matrices of the selected NAOs and **S**_*ab*_ the analytical overlap matrix between the two basis sets, computed with PySCF.^24^ The singular values of **W**=**C**_*a*_^T^ **S**_*ab*_ **C**_*b*_ are the cosines of the principal angles between the subspaces, and

3 $$\Omega =\frac{∥W{∥}_{F}^{2}}{r}=\frac{1}{r}\sum_{i=1}^{r}\cos^{2}\theta_{i},,r=\min\left(d_{a},d_{b}\right)$$

measures their overlap. Here *i* labels the *r* principal angles (or, equivalently, the singular values of **W**). Ω = 1 if and only if the smaller subspace is contained in the larger. We report δ = 1 – Ω, the deviation from perfect nesting. Because the standard and frozen-NMB constructions do not provide layer labels, their comparison spaces were assembled from principal shell labels under the eligibility rule of eq 2, with dimensions matched to the SNAO levels. Two reference values calibrate the scale: occupied RHF spaces obtained in neighboring basis sets give Ω = 0.998–1.000, whereas random subspaces of the same dimensions give Ω ≈ 0.040.

For the correlation-energy analysis, the SNAO hierarchy was built from the unrelaxed MP2 density, which is also the density used to define the frozen natural orbitals (FNOs) with which it is compared.^6,7^ The two hierarchies therefore contain the same correlated one-particle information and differ only in the constraints used to organize it. This use of an MP2 density is a controlled comparison of representations, not a claim of a cost-free truncation protocol: the cost of generating the density is not included in the timings, and no net speedup over a full MP2 calculation is claimed here. A hierarchy built from the RHF density serves as a control (Supporting Information).

Frozen-core RI-MP2 energies, with automatically generated auxiliary bases, were evaluated in truncated virtual spaces derived from a fixed cc-pV7Z parent calculation. The NAOs of the retained levels were projected onto the orthogonal complement of the occupied orbitals, canonically orthonormalized to the intended dimension, and semicanonicalized in the Fock matrix. The remaining virtual orbitals were then excluded from the correlation treatment by an orbital-energy window, leaving the parent-basis RHF determinant unchanged. At each dimension, the FNO calculation retains the same number of leading natural virtual orbitals. The complete transformation and its validation against the corresponding built-in ORCA procedure, to 8 × 10^–9^ *E*_*h*_, are described in the Supporting Information.

The ring sequences use the same procedure at the cc-pV5Z parent: the shell-nested and FNO hierarchies are built from the RI-MP2 unrelaxed density, and the level and full-space energies are evaluated with RI–SCS–MP2. For reference, each level-truncated ring calculation took about 4% of the wall time of the full cc-pV5Z calculation at the MP2 stage, where the integral transformation dominates. This timing describes the cost of evaluating an already generated hierarchy and does not include construction of the MP2 density.

For the 32-molecule set the same protocol was applied in a cc-pV5Z parent basis. The RHF reference and the unrelaxed RI-MP2 density were obtained in cc-pV5Z; the shell-nested hierarchy and the FNO sequence were built from this density with runsnvo, and frozen-core RI-MP2 energies were evaluated in the level-truncated spaces *L*_2_ and *L*_3_ and in the FNO spaces of the same dimensions. Level *L*_4_ is the complete cc-pV5Z virtual space, so the two sequences share their last member.

The retained dimensions make the truncation explicit. Across the 32-molecule set, the full virtual space contains 173–591 orbitals. Levels *L*_1_, *L*_2_, and *L*_3_ retain 19–60, 51–162, and 101–333 orbitals, respectively, corresponding to 9.7–12.0%, 26.8–30.3%, and 55.9–58.9% of the full virtual space. For benzene, pyrrole, and furan the corresponding dimensions are 93/243/489 of 855 (10.9/28.4/57.2%), 77/202/407 of 712 (10.8/28.4/57.2%), and 72/188/377 of 705 (10.2/26.7/53.5%). The FNO spaces are rank-matched to these dimensions.

Full-space RI-MP2 energies in cc-pVTZ, cc-pVQZ, and cc-pV5Z at the same geometries provide the primary external reference: the two-point *n* ^–3^ estimate of Helgaker and co-workers^25^ from the cc-pVQZ and cc-pV5Z energies, denoted *E*_CBS_^(Q,5)^. Because the internal and primary external estimates use different analytic forms, the comparison was also repeated against the *n* ^–3^ estimate from cc-pVTZ and cc-pVQZ and, on a like-for-like basis, against the same three-point exponential form applied to the full cc-pVTZ, cc-pVQZ, and cc-pV5Z energies.

Formal limits were obtained from the empirical three-parameter exponential form

4 $$E\left(n\right)=E_{\infty }+A\exp\left(-bn\right)$$

used previously for regular systematic sequences of wave functions and basis sets.^26^ Here *n* is only an equally spaced sequence coordinate: the hierarchy-level index for an internal fit and the cardinal number for an external basis-set fit. We do not identify *n* with an atomic angular momentum, a wave function norm, or a formally derived asymptotic variable. The model is used as a diagnostic of whether a nested sequence has acquired a regular limiting form.

For the cc-pV7Z parent, the internal fits use sliding three-level windows; for the cc-pV5Z parent they use levels 2–4, whose shell content corresponds to the cc-pVTZ-cc-pV5Z cardinals. A three-point fit is determined exactly; a decaying solution with *b* > 0 exists only when the energy increments decrease along the sequence. Windows with increasing increments are therefore reported as not converged rather than assigned an artificial limit. Model dependence was tested with five additional forms: the *n* ^–3^ expression of Helgaker and co-workers,^25^ a free power law, a two-term *n* ^–3^ + *n* ^–5^ expression, a shifted power law, and the root-exponential form of Zhong, Barnes, and Petersson^27^ as a function of the active-virtual count. The qualitative ordering of the hierarchies is unchanged (Supporting Information). The full-space end point provides an exact internal check: level 6 recovers the complete cc-pV7Z correlation energy, and level 4 the complete cc-pV5Z energy, to all reported digits for every construction.

The algebraic SNAO recursion does not require a correlation-consistent parent basis. Its interpretation as a cardinal-like extrapolation coordinate does. All numerical claims about cross-basis nesting and level extrapolation in the present work are therefore restricted to first-row cc-pV*n*Z sequences. For basis families with a different contraction or polarization schedule, the levels remain nested by construction, but their relation to a family index and to a CBS limit must be established separately. The present data do not distinguish how much of the observed regularity is intrinsic to the SNAO recursion and how much is inherited from the parent correlation-consistent family.

Complete population tables, radial sequences, subspace overlaps, truncated-space correlation energies, and the analysis scripts are supplied as Supporting Information.

## Results and Discussion

### Natural Populations Remain Unchanged

Reordering the virtual space is useful only if it leaves the chemically dominant NAO picture intact. Figure 2 shows the natural population analysis (NPA) charge of the heavy atom in the five molecular hydrides as a function of basis-set cardinal for the three constructions and RHF densities. The values agree to within 0.0005 *e* at cc-pVDZ and within 0.008 *e* at cc-pV7Z; the same bounds hold for the CCSD densities and for the three rings. The shell-nested construction therefore changes the organization of the Rydberg and virtual space without materially changing the NMB-dominated natural populations.

**Figure 2:**
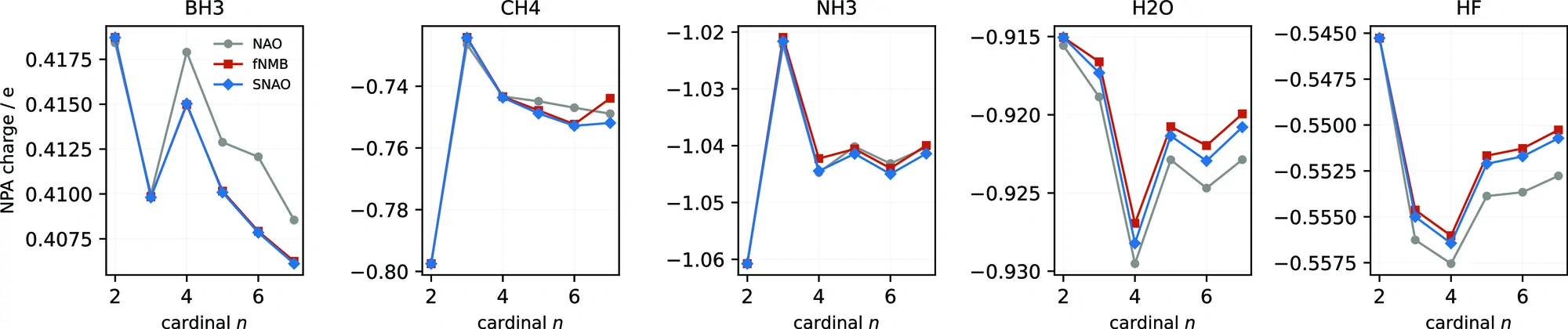
NPA charge of the heavy atom in five hydrides as a function of the cc-pV*n*Z cardinal (*n* = 2–7), for the standard, frozen-NMB, and shell-nested NAO constructions (RHF densities).

### Radial Occupations Recover Shell Order

A shell hierarchy should become progressively less occupied along each angular-momentum channel: for example, the first correlating *d* function of fluorine should carry more density than the second. An adjacent inversion is recorded when two successive radial functions of the same (*A*, *l*) channel, ordered by their principal labels, satisfy *n*_*k*+1_(*A*, *l*)–*n*_*k*_(*A*, *l*) > 1.5 × 10^–5^ *e*. The standard NAO construction gives monotonic radial sequences throughout the six-system first-row benchmark (Table 1); its sensitivity near the CBS limit lies instead in the AO-coefficient expansion discussed in ref 4. Frozen-NMB was designed to define the NMB before the Rydberg complement, not to impose an order on that complement, and its one-step Rydberg cycle accordingly gives 52 (RHF) and 33 (CCSD) adjacent inversions. SNAO selects the largest residual occupation in each channel at every cycle and therefore establishes a nonincreasing sequence before the final within-layer orthogonalization.

**Table 1:** Number of Adjacent Pairs with Inverted Order in Per-Channel Radial Occupation Sequences (Increase Larger Than 1.5 × 10^–5^ *e*) for the Six-System First-Row Benchmark (Five Hydrides Plus Ne; 1040 Adjacent Pairs per Entry), Together with the Largest Inversion

	RHF		CCSD	
construction	count	max/*e*	count	max/*e*
standard	0	–	0	–
frozen-NMB	52	6.0 × 10^–4^	33	1.1 × 10^–3^
shell-nested	46	6.4 × 10^–4^	5	3.8 × 10^–4^

For the correlated densities, this order is almost completely retained: only five of 1040 adjacent pairs are inverted, all by less than 4 × 10^–4^ *e* and all in hydrogen channels. These small differences are introduced by the within-layer orthogonalization described under property (ii). For RHF densities, frozen-NMB and SNAO give similar counts (52 and 46), confined to channels with occupations of order 10^–5^ *e*, where the SCF density carries essentially no information on the radial order. Figure 3 illustrates the distinction for the fluorine channels of HF at cc-pV7Z: the frozen-NMB tail becomes nonmonotonic, whereas the SNAO sequence decreases throughout.

**Figure 3:**
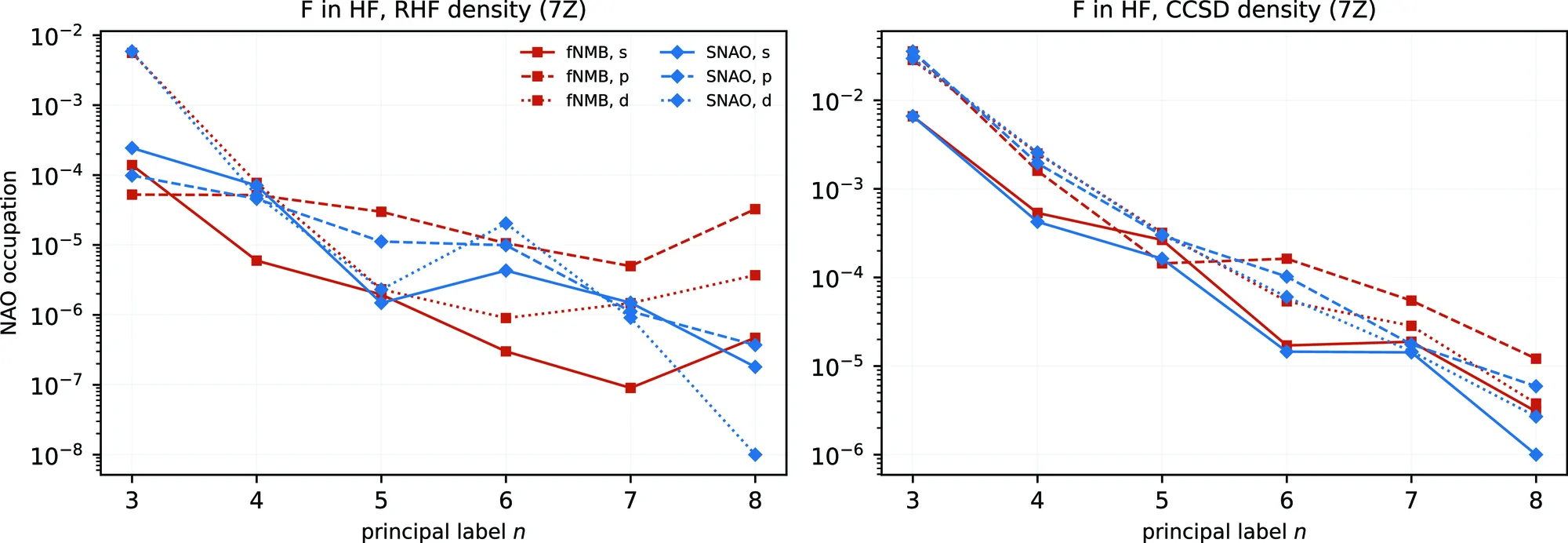
Radial occupation sequences of the fluorine *s*, *p*, and *d* channels in HF at cc-pV7Z, for the frozen-NMB and shell-nested constructions and for RHF and CCSD densities.

### Hierarchy Levels Converge Across Basis Sets

A shell label is transferable only if it identifies the same physical subspace as the basis is enlarged. Figure 4 reports the level-resolved deviation δ = 1 – Ω, eq 3, between levels obtained in neighboring basis sets, averaged over the molecular systems.

**Figure 4:**
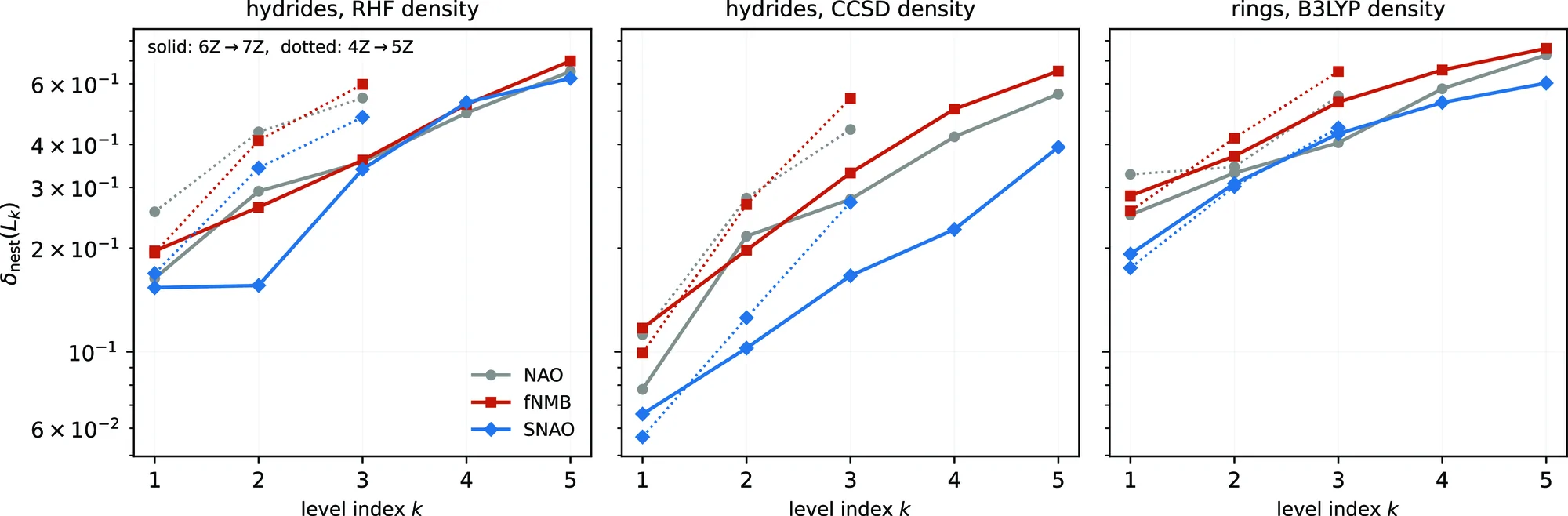
Deviation from perfect nesting, δ = 1 – Ω, for level *L*_*k*_ constructed in neighboring basis sets (solid: cc-pV6Z vs cc-pV7Z; dotted: cc-pVQZ vs cc-pV5Z), averaged over the molecular systems. Left to right: hydrides/RHF, hydrides/CCSD, and rings/B3LYP. For the standard and frozen-NMB constructions, the comparison spaces are principal-label shell sets of the same dimension as the corresponding SNAO level, assembled by the procedure described in Computational Methods.

Three trends emerge. First, SNAO gives the smallest deviation in every panel and at every depth, with the difference becoming larger for the higher levels. For the CCSD densities at cc-pV6Z versus cc-pV7Z, δ for *L*_3_ is 0.17 for SNAO, compared with 0.28 for the standard construction and 0.33 for frozen-NMB; at *L*_5_ the corresponding values are 0.39, 0.56, and 0.65. Higher levels change more strongly with the basis for every construction, but the SNAO subspaces change least.

Second, the SNAO deviations decrease systematically as the basis family is enlarged (dotted versus solid curves). The principal-label shell sets of the other constructions continue to change appreciably even between cc-pV6Z and cc-pV7Z; they were not designed to define nested virtual-space levels. Third, all three constructions coincide for the Ne atom to the reported precision: δ decreases from 7.5 × 10^–2^ for cc-pVDZ-cc-pVTZ to 2.2 × 10^–5^ for cc-pV6Z-cc-pV7Z. Without interatomic orthogonalization, each method reduces to channel-diagonal atomic eigenproblems that converge at the intrinsic rate of the basis family, although the radial functions can still be assigned to levels in different orders. The differences in Figure 4 therefore arise from the molecular coupling between atomic spaces. For all constructions, the small bases remain preasymptotic: δ ≈ 0.15–0.4 already for *L*_1_ at cc-pVDZ-cc-pVTZ. Quantitative transferability emerges from about cc-pVQZ onward.

This result should not be read as a basis-family independent theorem. The SNAO recursion guarantees nesting within one parent calculation, but the cross-cardinal similarity measured here may partly reflect the radial and angular schedule built into the cc-pV*n*Z family. A def2, pc-*n*, or ANO sequence has a different contraction and polarization history and need not support the same mapping between *L*_*k*_ and a family index. The present numerical evidence is therefore restricted to first-row correlation-consistent parent spaces; testing whether an equally regular coordinate emerges in other basis families is a separate question.

### Atomic Character of the Shell Layers

Figure 5 provides a real-space view of the first two carbon layers in benzene, with the valence 2*p*_3_ NAO as a reference. The level-1 functions are compact, single-node correlating orbitals with ANO-like radial forms. Level 2 repeats the angular families with one additional radial node and a more diffuse envelope. The images therefore give a direct chemical picture of the subspaces measured in the cross-basis nesting analysis: each level adds a complete, atom-centered shell of correlating functions.

**Figure 5:**
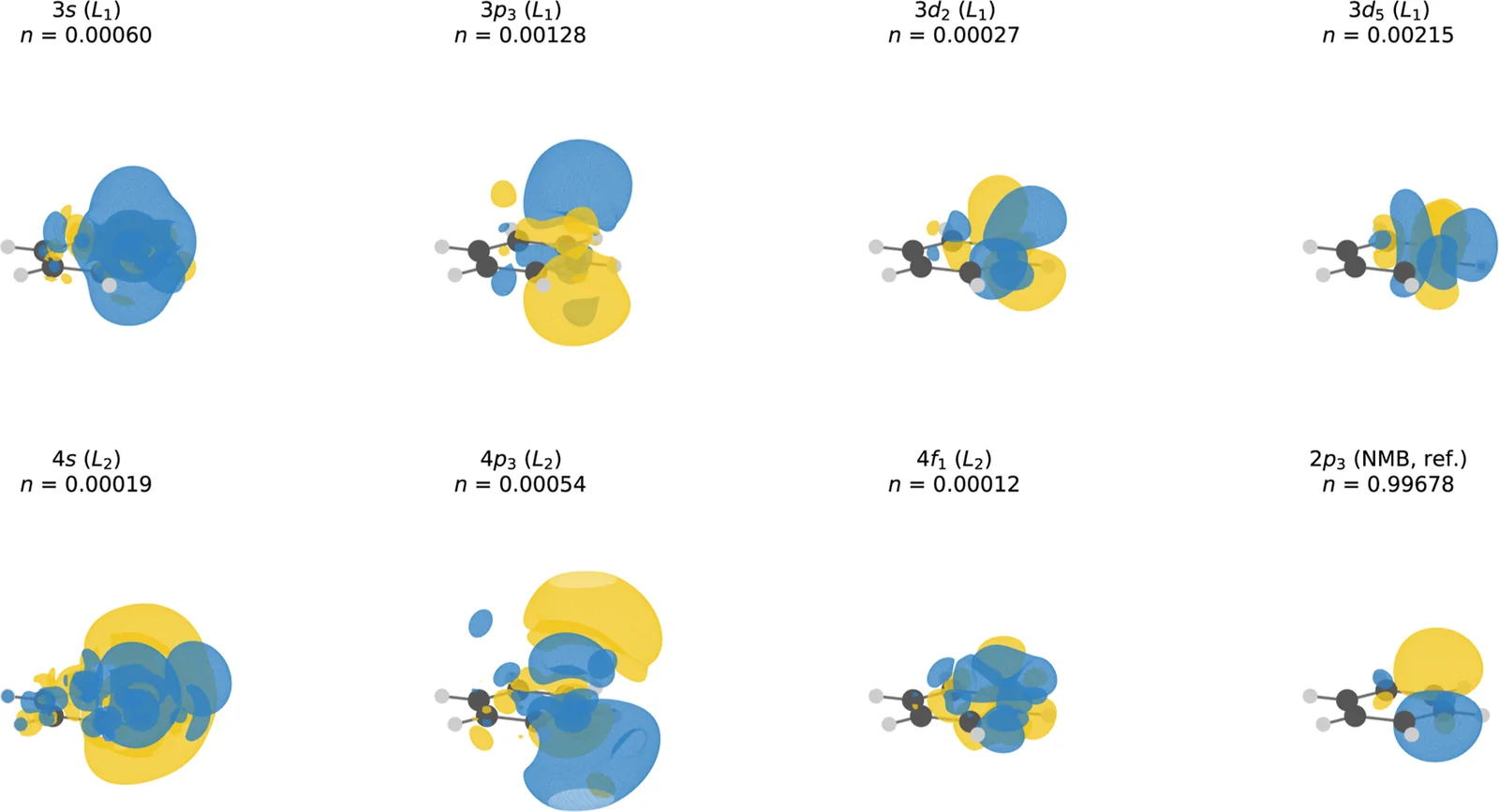
Representative carbon SNAOs in benzene (B3LYP/cc-pV5Z density; isovalue ±0.03). Top: level-1 functions (3*s*, 3*p*_3_, 3*d*_2_, and 3*d*_5_). Bottom: level-2 functions (4*s*, 4*p*_3_, and 4*f*_1_) together with the valence 2*p*_3_ NAO for reference. Occupations *n* are given in electrons.

### Compression and Extrapolation are Different Tasks

A virtual-space hierarchy can be judged in two distinct ways: by how much correlation energy it recovers at a fixed number of virtual orbitals and by how regularly it approaches a limiting space. FNO is designed for the first task. At a chosen dimension it retains the leading natural virtual orbitals of the MP2 density and is therefore expected to recover more correlation energy than a representation constrained to complete atomic shells. SNAO deliberately imposes the second kind of structure: its members form a nested sequence of atom- and shell-labeled spaces.

Building both hierarchies from the same MP2 density isolates the effect of that organization. The comparison is not intended as a demonstration of a cheaper way to obtain the MP2 density. Rather, it asks what is gained and lost when identical correlated information is arranged either for maximum fixed-rank recovery or for a transferable shell progression. The principal-label shell sets from the standard and frozen-NMB constructions provide two additional sequences of the same dimensions.

A further distinction from conventional CBS convergence is that the occupied reference does not change along the SNAO sequence. All levels are projected against the same parent-basis RHF occupied space and differ only in the virtual directions admitted to the correlation treatment. The increments therefore measure the radial and angular correlation freedom added to a common reference, without simultaneously changing the Hartree–Fock solution.

As expected, FNO recovers more correlation energy at a given number of virtual orbitals (Figure 6a). At level 1, SNAO recovers 65–74% of the full-space correlation energy, compared with 75–85% for FNO. The effect of the shell constraint decreases rapidly with depth: the difference falls from 7–15 percentage points at level 1 to about one point at level 4. The principal-label shell sets from the standard and frozen-NMB constructions recover slightly less and are nearly indistinguishable from each other, whereas layer-wise freezing moves the SNAO recovery closer to FNO. For Ne, all four sequences coincide exactly, as expected when the density and the basis are both organized by one atomic center.

**Figure 6:**
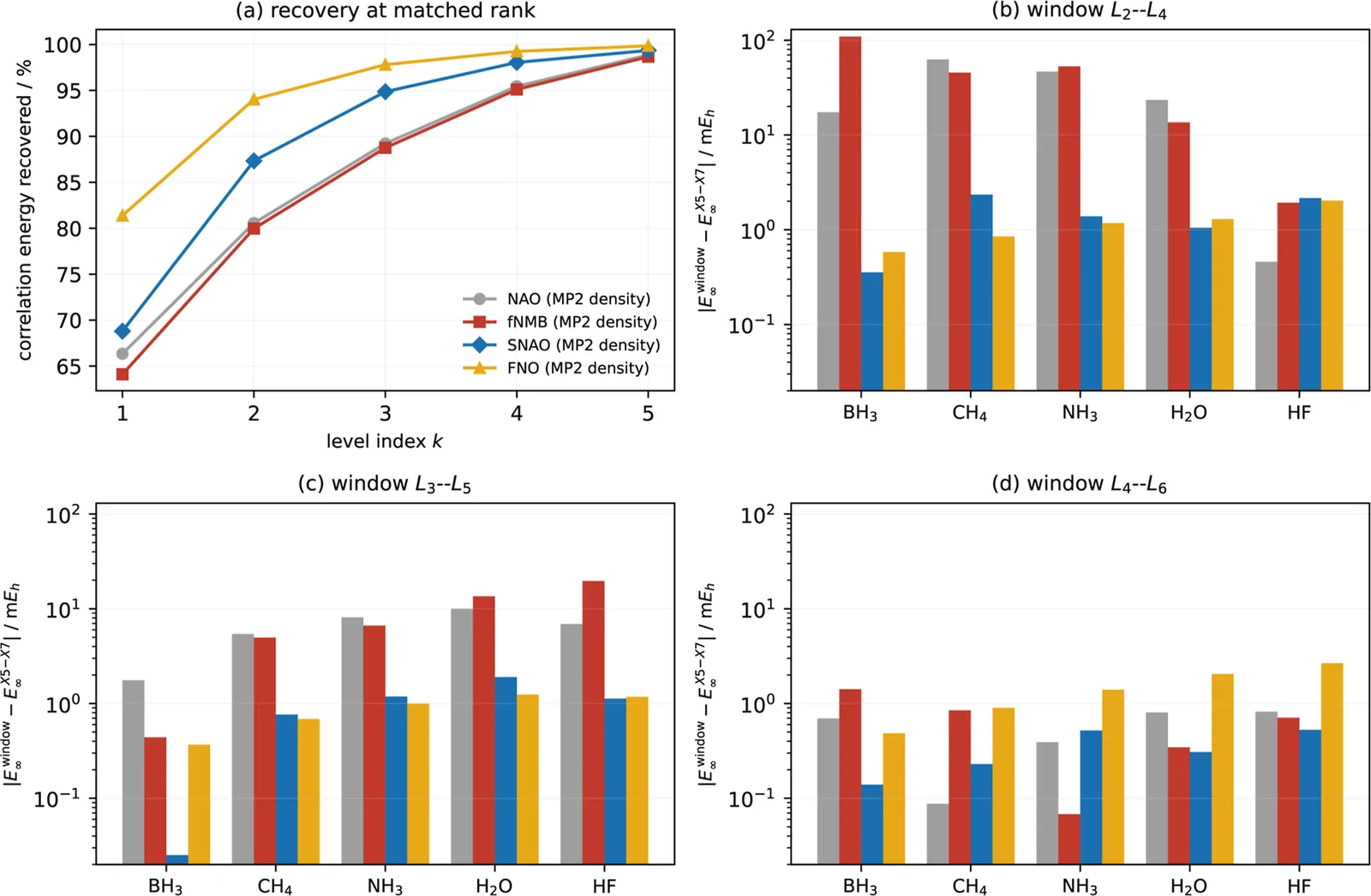
Four virtual-space sequences of matched dimensions built from the same unrelaxed MP2 density in a cc-pV7Z parent calculation: principal-label shell sets from the standard and frozen-NMB constructions, SNAO levels, and FNOs. (a) Correlation energy recovered at each level, averaged over the five molecules. (b–d) Absolute error of the empirical three-point exponential limit, eq 4, relative to the corresponding formal limit of the full cc-pV5Z-cc-pV7Z sequence (logarithmic scale). From the middle to the deepest window, the FNO extrapolation error increases for every molecule, whereas the SNAO error decreases for four and remains below 0.15 m*E*_*h*_ for the fifth. Ne is omitted because all four sequences coincide level by level to the reported precision.

The extrapolated limits show the complementary behavior (Figure 6b–d). The principal-label sequences become regular only near saturation: their intermediate three-level fits miss the reference limit by as much as 63 and 109 m*E*_*h*_, and only the deepest window reduces the error to 0.07–1.4 m*E*_*h*_. SNAO and FNO both give few-milli hartree errors in the intermediate windows and approach the milli hartree scale in the next window. Their behavior then separates. In the deepest window, the SNAO limits lie 0.14–0.53 m*E*_*h*_ from the formal cc-pV5Z-cc-pV7Z limit, whereas the FNO errors are 0.48–2.7 m*E*_*h*_, larger by factors of 2.7–6.6 for every molecule. Although the last point in the deepest FNO fit is the full parent-space energy, its extrapolation error increases from the preceding window for all five systems. The ordering persists for all six extrapolation forms tested, and the SNAO limit is also the least model-dependent: its median error varies from 0.25 to 0.62 m*E*_*h*_, compared with 1.1–2.3 m*E*_*h*_ for FNO. The RHF-density control spans 0.2–166 m*E*_*h*_ (Supporting Information), showing that shell order alone is not sufficient to rank correlation-energy contributions.

The energy gap between the matched-rank FNO and SNAO calculations is the finite-space price of imposing a fixed atom- and shell-resolved order. That price buys a different numerical property. FNO supplies the better individual point, whereas SNAO supplies the more regular sequence. A less optimal finite subspace can therefore give a more accurate estimate of the limiting energy when its convergence coordinate is physically defined and numerically stable. Maximum recovery at one dimension and reliable extrapolation through a sequence are distinct objectives.

The hierarchy also offers an interpretive use that is absent from a purely global rank threshold. With a localized occupied reference, the incremental correlation recovered on adding each SNAO layer can be resolved for an occupied orbital, an orbital pair, or a chemically defined fragment. Lone pairs, σ and π bonds, and delocalized regions need not exhaust their radial and angular correlation freedom at the same layer. Their layer-resolved saturation profiles could therefore guide adaptive local virtual spaces with domain-dependent shell depth. This capability follows from the present nested construction, although no domain decomposition is performed in the current benchmarks.

Finally, the construction of the hierarchy must be distinguished from the source of the correlated information used to order it. The present study uses a molecular MP2 density because it provides the same input for SNAO and FNO and hence a controlled comparison. Identifying cheaper or transferable sources of correlation-sensitive atomic information is a natural extension, but it is not required for the formal hierarchy or for the extrapolation tests established here.

The same analysis extends to larger molecules. For the three rings of the cross-basis nesting benchmark, SCS-MP2 level sequences were computed at the cc-pV5Z parent (up to 876 basis functions; RI throughout, hierarchies from the MP2 density, matched dimensions; Table 2). The recovered fractions are transferable: level 1 recovers 70–72% of the full–parent correlation energy in all three rings, level 2 88–89%, and level 3 96–97% (FNO: 82–83, 95, and 99%), so a level index carries the same meaning from one ring to the next, the energetic counterpart of the subspace transferability demonstrated above. The two principal-label sequences built from the same density at the same dimensions trail the shell-nested one by 3–12 percentage points at every level of every ring (standard 64–65, 80–81, and 93–94%; frozen-NMB 63–65, 76–77, and 91–92%), reproducing the hydride ordering in systems an order of magnitude larger. The level-4 calculation reproduces the full cc-pV5Z energy to all digits. Beyond the recovered fractions, a virtual space organized by atom and shell has a structure that a threshold-defined molecular space does not: every level is a complete set of atom-centered correlating shells, so a truncated space, and any quantity evaluated in it, can be resolved by atom, by angular momentum, and by radial node count. The present work uses the cross-basis subspace overlaps and the level extrapolation below. The same labels also make the hierarchy suitable for comparing how rapidly different localized occupied domains exhaust their correlation freedom.

**Table 2:** SCS-MP2 Level Sequences for the Three Rings (RI, Cc-pV5Z Parent): Correlation Energy Recovered per Level (%) for the Four Hierarchies of Matched Dimensions Built from the Same MP2 Density

system		standard	frozen-NMB	shell-nested	FNO
benzene	*L*_1_	64.1	64.5	71.6	82.7
(*n*_AO_ = 876)	*L*_2_	80.1	76.5	88.9	95.4
	*L*_3_	93.3	91.5	96.5	99.1
pyrrole	*L*_1_	64.2	63.3	70.7	82.4
(*n*_AO_ = 730)	*L*_2_	80.7	76.6	88.1	95.3
	*L*_3_	93.5	91.5	96.2	99.1
furan	*L*_1_	65.1	63.0	70.0	81.5
(*n*_AO_ = 723)	*L*_2_	81.4	76.3	88.1	95.0
	*L*_3_	93.6	91.3	96.1	99.1

### Level Extrapolation within a Single Basis Set

The hydride analysis used a cc-pV7Z parent so that internal three-level limits could be compared with an external family limit. The question that arises in practice is whether the SNAO levels of one finite parent calculation can estimate the basis-set limit of the correlation energy while the occupied RHF reference remains fixed. We address it for the 32-molecule set in cc-pV5Z. Levels L_2_, *L*_3_, and *L*_4_ have the shell content of the cc-pVTZ, cc-pVQZ, and cc-pV5Z virtual spaces, and *L*_4_ is the complete parent space. The FNO sequence is built from the same MP2 density at the same three dimensions, so the two sequences share their last member and differ only in the two lower ones. The primary reference is the two-point *n* ^–3^ estimate from the full cc-pVQZ and cc-pV5Z energies, *E*_CBS_^(Q,5)^. The same conclusions are checked against an *n* ^–3^ estimate from cc-pVTZ and cc-pVQZ and against a like-for-like three-point exponential fit to the full cc-pVTZ, cc-pVQZ, and cc-pV5Z energies. All energies, dimensions, and signed deviations are tabulated in the Supporting Information.

The recovery pattern of the hydrides recurs (Table 3): FNO recovers more of the parent correlation energy at both intermediate levels, 94.9 against 90.1% at *L*_2_ and 98.9 against 96.9% at *L*_3_. The extrapolated limits behave differently (Figure 7). The SNAO limits deviate from *E*_CBS_^(Q,5)^ by 3.2 m*E*_*h*_ on average (median 2.3), with both signs represented and no systematic offset; the largest deviations, 7–10 m*E*_*h*_, occur for the fluorine- and oxygen-rich molecules CO_2_, OF_2_, F_2_, oxirane, N_2_O, and CH_2_F_2_. The FNO limits deviate by 13.1 m*E*_*h*_ on average, and the deviation is positive for all 32 molecules: every FNO limit recovers less correlation energy than the reference, by 7 to 23 m*E*_*h*_. The SNAO limit is the closer one in every molecule, by a median factor of 4.8. The requirement of decreasing increments defined in Computational Methods is met by both sequences in all cases, so the difference does not arise from ill-conditioned fits.

**Table 3:** Level Extrapolation for the 32-Molecule Set in Cc-pV5Z: Correlation Energy Recovered at Levels 1–3 (% of the Full Cc-pV5Z Value, Mean Over Molecules) and Deviation of the Three-point Limit from the Two-Point Cc-pV­(Q,5)­Z Estimate (m*E*_*h*_) for the Windows of Levels 1–3 and 2–4^a^

	shell-nested	FNO
recovery at *L*_1_/%	71.8	81.7
recovery at *L*_2_/%	90.1	94.9
recovery at *L*_3_/%	96.9	98.9
window *L*_1_–*L*_3_		
mean absolute deviation	10.3	11.6
median absolute deviation	10.0	12.0
signed range	–1.5 to +21.1	+6.1 to +17.8
molecules with the closer limit	19	13
window *L*_2_–*L*_4_		
mean absolute deviation	3.2	13.1
median absolute deviation	2.3	13.8
maximum absolute deviation	10.1 (CO_2_)	22.6 (CH_2_F_2_)
signed range	–10.1 to +9.6	+7.1 to +22.6
molecules with the closer limit	32	0
*n* ^–3^ from (T,Q): MAD; closer	3.2; 30	9.5; 2
exponential (T,Q,5): MAD; closer	4.9; 28	7.5; 4

a The last two rows give the mean absolute deviation (MAD) of the *L*_2_–*L*_4_ limit and the number of molecules in which the given limit is the closer one for two alternative reference estimates.

**Figure 7:**
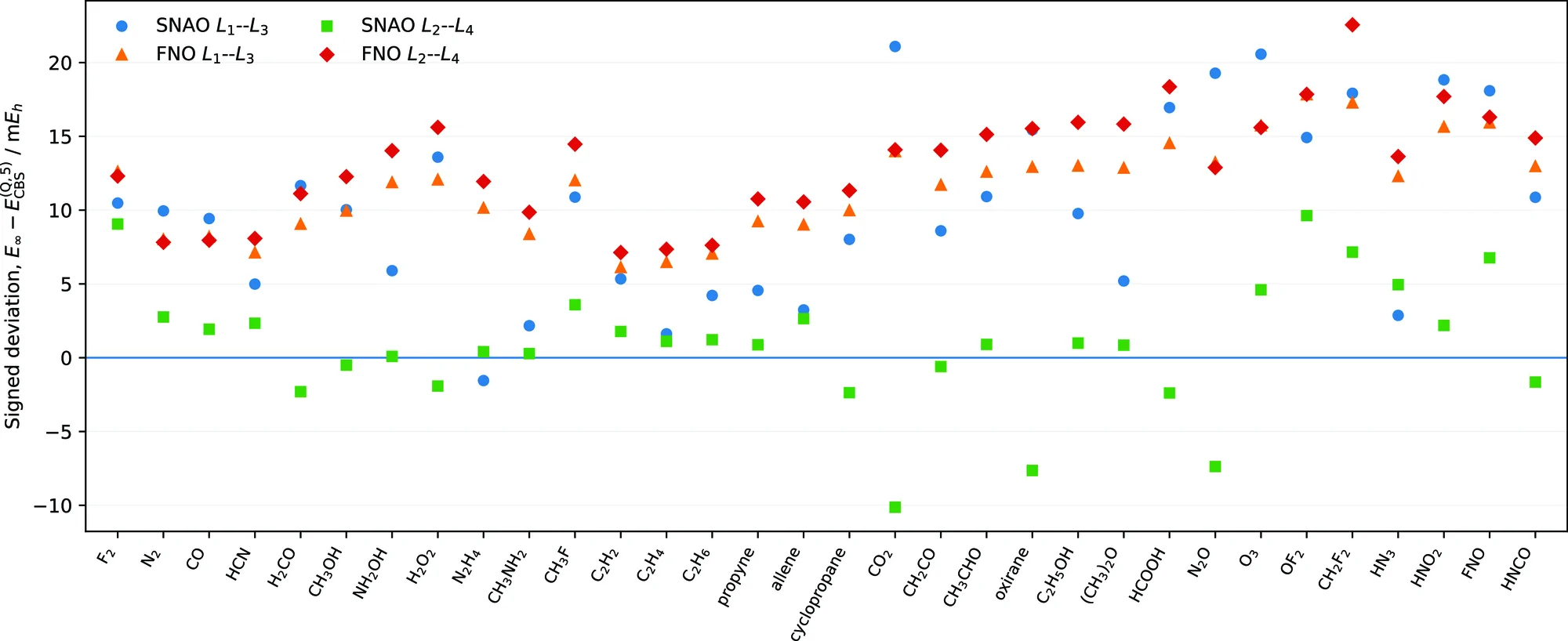
Signed deviation Δ = *E*_∞_–*E*_CBS_^(Q,5)^ of the empirical three-point exponential limit from the two-point cc-pV­(Q,5)­Z estimate for 32 molecules in a cc-pV5Z parent basis. Negative values indicate a limit below the reference (more negative correlation energy), and positive values a limit above it. The four series correspond to the *L*_1_–*L*_3_ and *L*_2_–*L*_4_ windows of the SNAO and rank-matched FNO hierarchies. The plotted values are those tabulated in Supporting Information Tables S16 and S17.

The sign pattern identifies the mechanism. An occupation-ordered sequence that already recovers 99% of the parent energy at its second member has nearly exhausted the parent space, and its rapidly shrinking increments describe convergence toward the finite cc-pV5Z energy; the exponential model reports that limit correctly and places it 7–23 m*E*_*h*_ above the basis-set estimate. The increments of the shell-nested sequence are instead those of the shell progression cc-pVTZ, cc-pVQZ, cc-pV5Z, and their limit is the limit of that progression. The same three-point exponential fit applied to the full cc-pVTZ, cc-pVQZ, and cc-pV5Z energies deviates from the two-point estimate by 5.6 m*E*_*h*_ on average (Supporting Information), a model-to-model difference of the same order as the SNAO deviation; the level sequence of a single cc-pV5Z calculation therefore reproduces the two-point estimate as closely as the three full-space calculations do. The conclusion does not depend on the reference chosen. Against the *n* ^–3^ estimate from cc-pVTZ and cc-pVQZ, the SNAO limit is the closer one in 30 of 32 molecules; against the exponential fit to the three cardinals, in 28 of 32 (Table 3). The size of the difference varies with the reference, its direction does not.

The window of levels 1–3, which has the shell content of the cc-pVDZ, cc-pVTZ, and cc-pVQZ spaces, shows where this behavior sets in (Figure 7a). Here the two sequences are not distinguished: the SNAO limits deviate from *E*_CBS_^(Q,5)^ by 10.3 m*E*_*h*_ on average and the FNO limits by 11.6 m*E*_*h*_, both almost always with a positive sign, and the SNAO limit is the closer one in 19 of 32 molecules. Both limits lie between the finite cc-pV5Z energy and the basis-set estimate. For the shell-nested sequence this is the preasymptotic behavior of the basis-set family itself: an extrapolation whose lowest member has cc-pVDZ shell content is no more reliable inside one basis set than the corresponding two-point cc-pV­(D,T)­Z extrapolation is between basis sets, and the cross-basis nesting measurements place the onset of quantitative transferability at about cc-pVQZ. The two sequences separate in the direction of their response to a deeper window. Moving from levels 1–3 to levels 2–4, the SNAO deviation decreases in 31 of 32 molecules, from 10.3 to 3.2 m*E*_*h*_ on average, whereas the FNO deviation increases in 27 of 32, from 11.6 to 13.1 m*E*_*h*_: the shell-nested sequence converges toward the basis-set limit as its window deepens, the occupation-ordered sequence toward the finite parent. The same window-to-window pattern was observed for the cc-pV7Z hydride sequences in the preceding compression–extrapolation analysis, where the FNO deviation grew from the middle to the deepest window for every molecule while the SNAO deviation shrank.

The three rings extend the observation to larger systems and to SCS-MP2 (Table 2). With the same *L*_2_–*L*_4_ window and the same cc-pV­(Q,5)­Z reference, the shell-nested limits deviate by 2.7, 7.0, and 8.1 m*E*_*h*_ for benzene, pyrrole, and furan, whereas the FNO limits fall short by 23.3, 21.8, and 23.9 m*E*_*h*_. In the window of levels 1–3 the ring sequences are already separated, the shell-nested limits deviating by – 1.7, −4.0, and +2.8 m*E*_*h*_ and the FNO limits by +19.7, +17.9, and +19.0 m*E*_*h*_; with 93 to 243 active virtual orbitals, the lowest levels of these larger molecules are further from the preasymptotic regime than the 19- to 60-dimensional *L*_1_ spaces of the small molecules.

## Conclusions

The frozen-NMB construction defines the chemically meaningful minimal space before the full Rydberg complement is restored. Applied recursively, the same operation turns that complement into shell-nested atomic orbitals: a sequence of frozen, atom-resolved layers that follows the shell schedule of a systematic correlation basis. The extension leaves natural populations essentially unchanged, restores the radial ordering of shell occupations for correlated densities, and produces level subspaces that become progressively more similar as the cc-pV*n*Z cardinal increases. For an isolated atom, the radial ordering is exact.

The conceptual gain is a common reference for studying correlation freedom. In a conventional CBS sequence, enlargement of the one-electron basis changes both the optimized Hartree–Fock solution and the virtual manifold. In an SNAO sequence, the parent-basis occupied RHF space is fixed and only the active virtual subspace is enlarged. Successive levels therefore separate the radial and angular degrees of freedom admitted to dynamic correlation from changes in the underlying reference.

The results also distinguish virtual-space compression from convergence of a sequence. At every matched dimension, FNO recovers more correlation energy, as expected for an occupation-optimized compressor. The FNO–SNAO energy difference is the finite-space cost of imposing a nested, atom- and shell-resolved order. That order produces a substantially more regular extrapolation coordinate. For 32 first-row molecules in a cc-pV5Z parent, the three-point limit of levels 2–4 differs from the two-point cc-pV­(Q,5)­Z estimate by 3.2 m*E*_*h*_ on average and with no systematic sign, whereas the rank-matched FNO limits fall short by 13.1 m*E*_*h*_ on average and in every molecule. The same distinction appears in the cc-pV7Z hydride sequences and in SCS-MP2 calculations for benzene, pyrrole, and furan. A better finite point and a better path to the limit are therefore not the same objective.

The shell-resolved coordinate also provides a platform for local analysis. With a localized occupied reference, the increment associated with each SNAO layer can be assigned to an occupied orbital, an orbital pair, or a fragment. Lone pairs, σ and π bonds, and delocalized domains may then be compared by the shell depth required to saturate their radial and angular correlation freedom. Such profiles offer a direct route to adaptive local virtual spaces with domain-dependent layer depth.

Two scope limitations are explicit. First, although the algebraic recursion can be applied to any atom-centered parent basis, the cardinal-like interpretation and extrapolation results have been demonstrated only for first-row correlation-consistent spaces; the extent to which the observed regularity is inherited from that family remains to be tested elsewhere. Second, the present MP2 density is a controlled common source of correlated information for the SNAO–FNO comparison, not a proposed cost-free generator of the hierarchy. The construction of the nested coordinate and the choice of a cheaper or transferable correlation-sensitive density are separate questions. The present work establishes the former and shows why its regularity is useful independently of the eventual source of the ranking information.

## Supplementary Material



## Data Availability

The runsnvo program (including the shell-nested construction, option –nao-algorithm snao, and the export of level-truncated virtual spaces) is freely available at https://aromaticity.uj.edu.pl/runsnvo. The density files, exported NAO sets, and analysis scripts supporting the reported results are available from the author upon reasonable request.

## Notes

Proof of monotonic radial selection; a matrix formulation of the shell-nested construction; complete radial occupation sequences and population tables; level-resolved and cumulative subspace overlaps (Ω, δ); truncated-space correlation energies and extrapolation tests; the complete energies, dimensions, and extrapolated limits for the 32-molecule set; the construction of level-truncated virtual spaces; a note on file-precision effects for deep Rydberg orbitals; the mapping to runsnvo command-line options; and benchmark geometries. Numerical data underlying all figures and tables are provided as tab-separated files (PDF)
