# Supplementary material for: Shell-Nested Atomic Orbitals: Restoring the Correlation-Consistent Hierarchy inside the Molecular Virtual Space
Source: J Phys Chem A. 2026 Oct 20;130(44):9009–19. doi: 10.1021/acs.jpca.6c06093 (PMC13746814; doi:10.1021/acs.jpca.6c06093)
Supplement: Supplementary file 1 [file jp6c06093_si_001.pdf]

Supporting Information

Shell-Nested Atomic Orbitals: Restoring the Correlation-Consistent Hierarchy inside the Molecular Virtual Space

Dariusz W. Szczepanik

K. Gumiński Department of Theoretical Chemistry, Faculty of Chemistry, Jagiellonian University, Gronostajowa 2, 30-387 Kraków, Poland.
E-mail: dariusz.szczepanik@uj.edu.pl

Table of contents

S1 Proof of the radial monotonicity property . . . S2
S2 Radial occupation sequences . . . S2
S3 Subspace-overlap tables . . . S2
S4 Level extrapolation for the 32-molecule set in cc-pV5Z . . . S11
S5 Matrix formulation of the shell-nested construction . . . S15
S6 Construction of the level-truncated virtual spaces . . . S15
S7 Precision limits of interchange formats for deep Rydberg orbitals . . . S16
S8 Terminology and program options . . . S17
S9 Geometries . . . S17

S1 Proof of the radial monotonicity property

Let $\mathbf{M} = \mathbf{SDS}$ be the occupation form of the main text, and let $\mathcal{C}_k$ denote the residual space from which cycle k selects: the orthogonal complement (in the $\mathbf{S}$ metric) of the natural minimal basis and of all layers frozen before cycle k. For a channel (A, l), let $\mathcal{C}_k(A, l) \subseteq \mathcal{C}_k$ be the span of the channel's residual radial functions, and let

$$n_k(A, l) \;=\; \max_{\substack{\mathbf{v} \in \mathcal{C}_k(A,l) \\ \mathbf{v}^\mathsf{T}\mathbf{S}\mathbf{v}=1}} \mathbf{v}^\mathsf{T}\mathbf{M}\,\mathbf{v} \tag{S1}$$

be the occupation of the vector selected from the channel in cycle k (step 2 of the construction keeps exactly the maximizer, computed on the m-averaged form).

Cycle k removes the selected vectors from the working space and adds nothing, so

$$\mathcal{C}_{k+1} \subseteq \mathcal{C}_k \qquad \text{and, channel-wise,} \qquad \mathcal{C}_{k+1}(A, l) \subseteq \mathcal{C}_k(A, l), \tag{S2}$$

because the maintained working copy of each channel's radial functions is only ever projected (never rotated between channels). The maximum of a fixed quadratic form over a shrinking admissible set cannot increase; therefore

$$n_1(A, l) \;\geq\; n_2(A, l) \;\geq\; n_3(A, l) \;\geq\; \ldots \tag{S3}$$

for every channel and for an arbitrary density matrix $\mathbf{D}$ (SCF or correlated), at the moment of selection. ■

Two remarks. (i) The within-layer occupation-weighted symmetric orthogonalization applied after selection forms linear combinations inside the layer subspace only; it preserves the layer span, but it can move occupation between members of the same layer that reside on different atoms and overlap appreciably. The final (post-orthogonalization) occupations can therefore violate the ordering by at most the amount this step moves. In the benchmark sets of the main text the violations are confined to nearly unoccupied channels and do not exceed 1×10^{-3} electrons (Tables S2–S3 below and Table 1 of the main text). (ii) For an isolated atom, functions on different atoms do not occur, symmetry-inequivalent channels of one atom are mutually $\mathbf{S}$-orthogonal, and the orthogonalization step reduces to the identity; the ordering is then exact, which the neon results of the main text confirm to all reported digits.

S2 Radial occupation sequences

Tables S1–S2 give the complete fluorine radial occupation sequences of the HF molecule at cc-pV7Z for both density types, and Table S3 the per-system statistics of ordering violations. An adjacent inversion means that two successive radial functions of the same (A, l) channel, ordered by their principal labels, satisfy $n_{k+1}(A, l) - n_k(A, l) > 1.5 \times 10^{-5}\, e$. The complete sequences for all systems, densities, cardinals, and constructions are provided as a machine-readable table (`shell_ladders.tsv`).

S3 Subspace-overlap tables

Tables S4–S9 give the deviation from perfect nesting, $\delta = 1 - \Omega$, for individual and cumulative hierarchy levels, averaged over the molecular systems of each benchmark set; Table S10 gives the calibration anchors. Tables S11–S13 support the main-text subsection "Compression and extrapolation are different tasks": per-level correlation-energy recovery for the shell-nested and FNO hierarchies built from the same MP2 density (S11), prediction of the full-parent correlation

energy from three-level windows, including the RHF-built control hierarchy (S12), and the deepest-window formal limits versus the external cc-pV5Z–cc-pV7Z limit (S13). Table S14 documents the model dependence of the window extrapolation across six analytic forms.

The exponential entry in Table S14 denotes the empirical three-parameter form of Eq. (4) of the main text applied to an equally spaced sequence index. It is not interpreted as a function of atomic angular momentum or wavefunction norm. Figure S2 traces the corresponding window sequences for the two MP2-density hierarchies. The deepest window gives the most accurate limit of the shell-nested sequence in four of the five molecules, whereas the FNO deviation increases from the L_3–L_5 to the L_4–L_6 window in all five—although the deepest FNO point is the exact full space. At the deepest window the shell-nested limit is closer to the external one in all five molecules, and Table S14 shows this ordering to be independent of the extrapolation model.

All cross-basis and extrapolation tests in this Supporting Information use the first-row cc-pVnZ family. The SNAO recursion itself can be applied to another atom-centred parent basis, but the identification of L_k with a cardinal-like coordinate depends on a systematic shell schedule. No basis-family-independent extrapolation claim is made for def2, pc-n, ANO, or other parent spaces.

A reference of a different kind was also tested: replacing the external family limit by the two-point n^{-3} complete-basis-set estimate from the cc-pV6Z and cc-pV7Z energies. Against this reference all four hierarchies acquire a common deviation of 2–9 mE_h that grows with the correlation energy of the system and nearly equalizes the hierarchies at the deepest window: the two-point estimate extrapolates beyond the finite parent family, so the comparison then measures the basis-set incompleteness of the cc-pV7Z family itself rather than the quality of the internal hierarchies. Internal and external limits are therefore compared with the same model throughout. Figure S1 shows the residual correlation energy along the hierarchies.

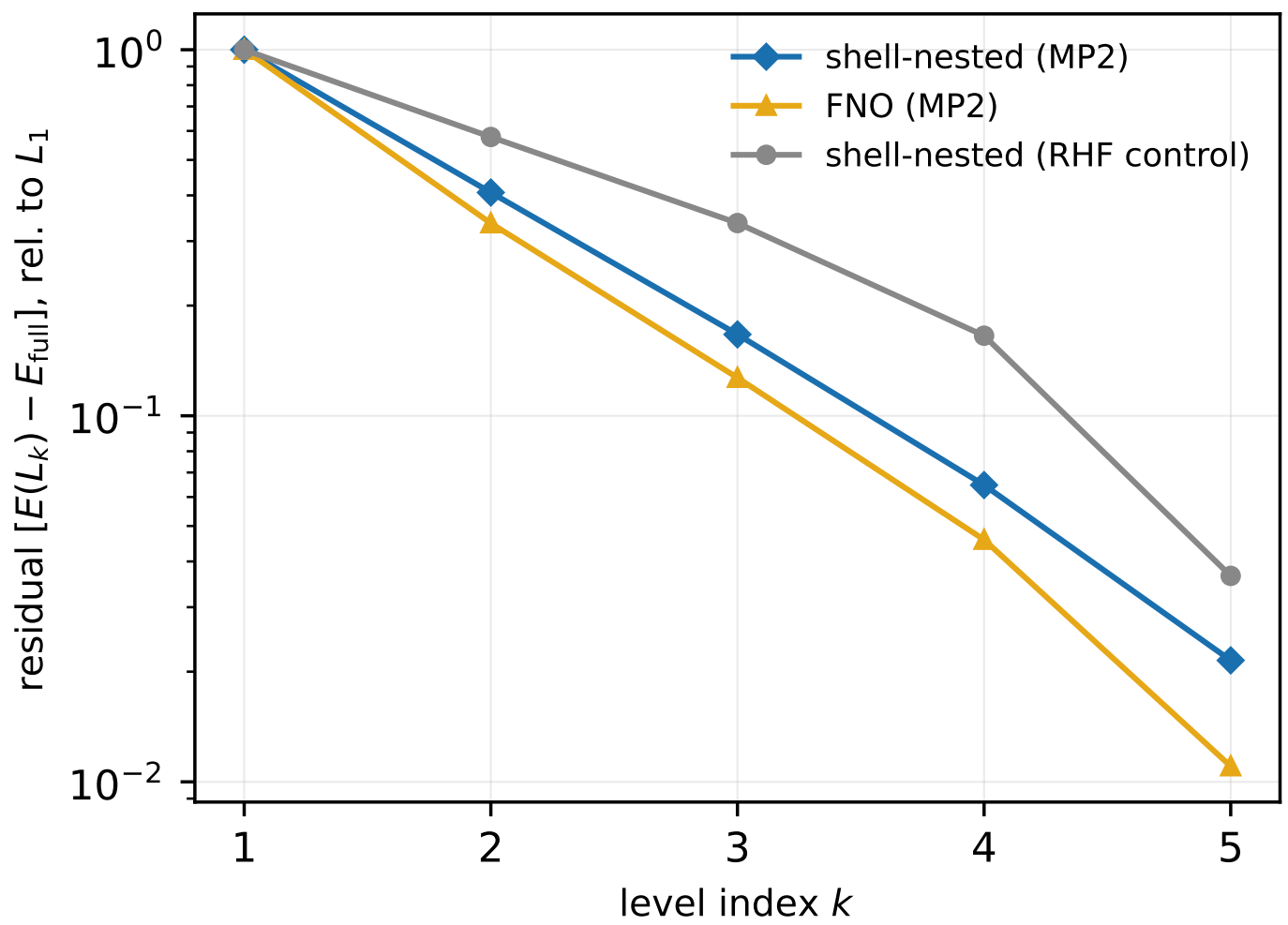

Figure S1. Residual correlation energy $E(L_k) - E_{\mathrm{full}}$ of the truncated RI-MP2 calculations, relative to level 1, averaged over the six systems (cc-pV7Z parent): shell-nested and FNO hierarchies from the MP2 unrelaxed density, and the RHF-built shell-nested control.

The complete per-system data, including the cc-pVnZ-vs.-cc-pV7Z pairs, the NMB rows, and all truncated-space RI-MP2 energies, are provided as machine-readable tables (`nesting_hydrides.tsv`, `nesting_rings.tsv`, `sanity_anchors.tsv`, `extrap_results.tsv`, `external_results.tsv`, `extrap_summary.tsv`), together with the analysis scripts.

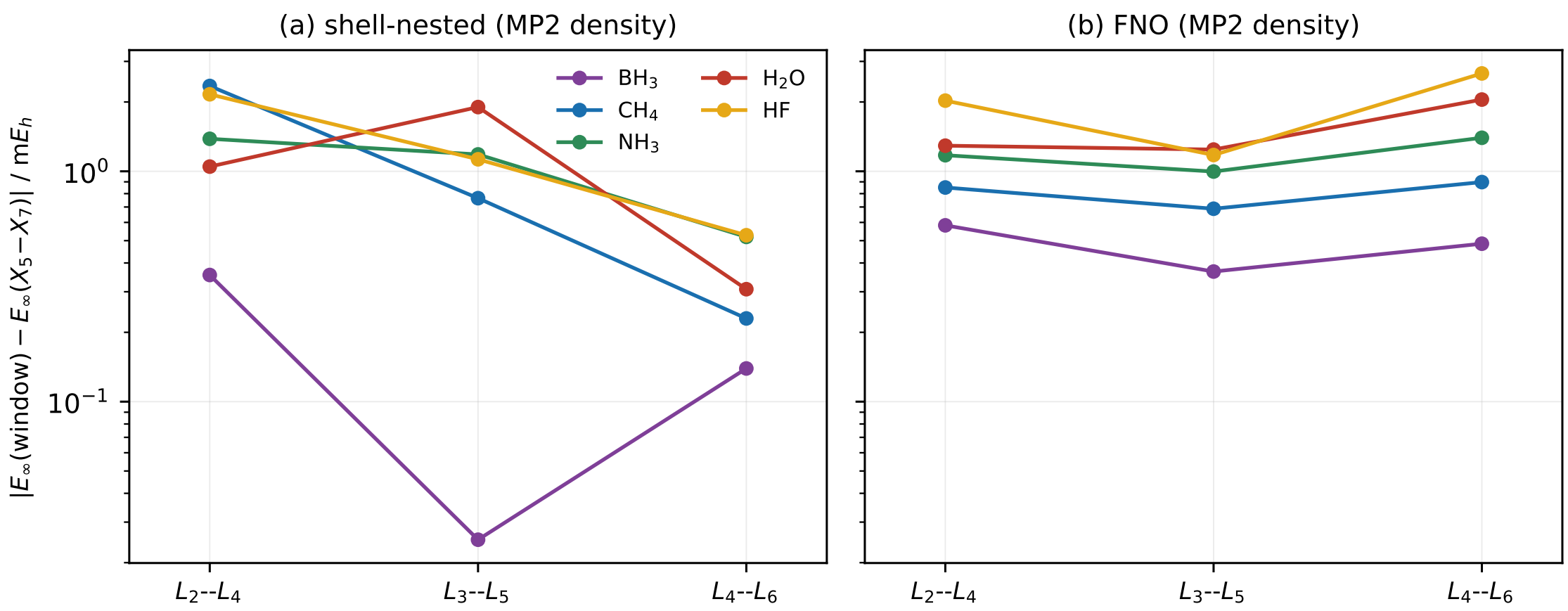

Figure S2. Absolute deviation of the internal window limit from the external cc-pV5Z–cc-pV7Z limit for the sliding three-level windows (empirical three-point exponential model on the level index, decreasing-increment requirement as in the main text), for the shell-nested (a) and FNO (b) hierarchies built from the same MP2 unrelaxed density at matched dimensions.

Table S1. Radial occupation sequences of the fluorine Rydberg channels in the HF molecule at cc-pV7Z (RHF density). Entries in electrons; inverted adjacent pairs are discussed in the main text.

Shell	standard	frozen-NMB	shell-nested
$3s$	0.0000061	0.0001387	0.0002436
$4s$	0.0000000	0.0000060	0.0000708
$5s$	-0.0000000	0.0000019	0.0000015
$6s$	-0.0000000	0.0000003	0.0000043
$7s$	-0.0000000	0.0000001	0.0000015
$8s$	0.0000000	0.0000005	0.0000002
$3p$	0.0003149	0.0000524	0.0000985
$4p$	0.0000765	0.0000517	0.0000457
$5p$	0.0000002	0.0000298	0.0000112
$6p$	0.0000000	0.0000106	0.0000099
$7p$	0.0000000	0.0000050	0.0000011
$8p$	-0.0000000	0.0000325	0.0000004
$3d$	0.0094341	0.0055943	0.0058822
$4d$	0.0000260	0.0000772	0.0000506
$5d$	0.0000084	0.0000023	0.0000023
$6d$	0.0000000	0.0000009	0.0000203
$7d$	0.0000000	0.0000015	0.0000009
$8d$	0.0000000	0.0000037	0.0000000

Table S2. Radial occupation sequences of the fluorine Rydberg channels in the HF molecule at cc-pV7Z (CCSD density). Entries in electrons; inverted adjacent pairs are discussed in the main text.

Shell	standard	frozen-NMB	shell-nested
$3s$	0.0067781	0.0065952	0.0066204
$4s$	0.0001895	0.0005349	0.0004249
$5s$	0.0000171	0.0002653	0.0001627
$6s$	0.0000043	0.0000171	0.0000145
$7s$	0.0000009	0.0000188	0.0000143
$8s$	0.0000002	0.0000031	0.0000010
$3p$	0.0369909	0.0356941	0.0356926
$4p$	0.0015059	0.0015997	0.0019433
$5p$	0.0002110	0.0001442	0.0002980
$6p$	0.0000332	0.0001629	0.0001023
$7p$	0.0000113	0.0000548	0.0000176
$8p$	0.0000033	0.0000121	0.0000059
$3d$	0.0313189	0.0285229	0.0296857
$4d$	0.0024820	0.0025121	0.0025753
$5d$	0.0002181	0.0003192	0.0003043
$6d$	0.0000440	0.0000537	0.0000603
$7d$	0.0000109	0.0000285	0.0000146
$8d$	0.0000020	0.0000038	0.0000027

Table S3. Inverted adjacent pairs in per-channel radial occupation sequences (occupation increase above $1.5 \times 10^{-5}\, e$), per system, over all Rydberg channels and cardinals (inverted/total).

	RHF			CCSD		
System	stand.	froz.	nest.	stand.	froz.	nest.
BH_3	0/215	12/215	20/215	0/215	4/215	0/215
CH_4	0/265	15/257	9/265	0/265	13/265	1/265
NH_3	0/215	10/215	11/215	0/215	5/215	0/215
H_2O	0/165	9/165	3/165	0/165	5/165	0/165
HF	0/115	6/115	3/115	0/115	6/115	4/115
Ne	0/65	0/65	0/65	0/65	0/65	0/65

Table S4. Deviation from perfect nesting, $\delta = 1 - \Omega$, of individual levels L_k built in neighbouring basis sets (hydrides, RHF densities; mean over systems).

K	pair	standard	frozen-NMB	shell-nested
1	2Z→3Z	0.3258	0.2660	0.2375
1	3Z→4Z	0.3558	0.2651	0.2466
1	4Z→5Z	0.2549	0.1937	0.1689
1	5Z→6Z	0.2180	0.1622	0.1403
1	6Z→7Z	0.1635	0.1962	0.1535
2	3Z→4Z	0.4298	0.4950	0.4036
2	4Z→5Z	0.4350	0.4107	0.3418
2	5Z→6Z	0.3805	0.2786	0.2751
2	6Z→7Z	0.2926	0.2628	0.1558
3	4Z→5Z	0.5460	0.5978	0.4802
3	5Z→6Z	0.4216	0.4527	0.4784
3	6Z→7Z	0.3555	0.3601	0.3388
4	5Z→6Z	0.6174	0.6811	0.5749
4	6Z→7Z	0.4938	0.5228	0.5301
5	6Z→7Z	0.6518	0.6990	0.6222

Table S5. Deviation from perfect nesting, $\delta = 1 - \Omega$, of individual levels L_k built in neighbouring basis sets (hydrides, CCSD densities; mean over systems).

K	pair	standard	frozen-NMB	shell-nested
1	2Z→3Z	0.2081	0.1812	0.1448
1	3Z→4Z	0.1187	0.1286	0.0989
1	4Z→5Z	0.1121	0.0991	0.0566
1	5Z→6Z	0.0691	0.0836	0.0310
1	6Z→7Z	0.0778	0.1172	0.0660
2	3Z→4Z	0.2717	0.3458	0.2032
2	4Z→5Z	0.2793	0.2677	0.1255
2	5Z→6Z	0.2557	0.2613	0.0959
2	6Z→7Z	0.2166	0.1973	0.1025
3	4Z→5Z	0.4421	0.5443	0.2718
3	5Z→6Z	0.3256	0.3818	0.1753
3	6Z→7Z	0.2773	0.3306	0.1663
4	5Z→6Z	0.5294	0.6125	0.3524
4	6Z→7Z	0.4211	0.5066	0.2267
5	6Z→7Z	0.5604	0.6532	0.3928

Table S6. Deviation from perfect nesting, $\delta = 1 - \Omega$, of individual levels L_k built in neighbouring basis sets (rings, B3LYP densities; mean over systems).

K	pair	standard	frozen-NMB	shell-nested
1	2Z→3Z	0.3332	0.2650	0.2412
1	3Z→4Z	0.4010	0.2024	0.1239
1	4Z→5Z	0.3276	0.2563	0.1752
1	5Z→6Z	0.2538	0.2489	0.1809
1	6Z→7Z	0.2498	0.2838	0.1923
2	3Z→4Z	0.4334	0.4920	0.3282
2	4Z→5Z	0.3441	0.4173	0.3018
2	5Z→6Z	0.3405	0.3130	0.2677
2	6Z→7Z	0.3306	0.3699	0.3080
3	4Z→5Z	0.5530	0.6513	0.4470
3	5Z→6Z	0.4785	0.5770	0.3835
3	6Z→7Z	0.4045	0.5310	0.4298
4	5Z→6Z	0.6910	0.7054	0.5523
4	6Z→7Z	0.5803	0.6579	0.5295
5	6Z→7Z	0.7277	0.7600	0.6027

Table S7. Deviation from perfect nesting, $\delta = 1 - \Omega$, of cumulative levels $L_1 \oplus \cdots \oplus L_K$ built in neighbouring basis sets (hydrides, RHF densities; mean over systems).

K	pair	standard	frozen-NMB	shell-nested
1	2Z→3Z	0.3258	0.2660	0.2375
1	3Z→4Z	0.3558	0.2651	0.2466
1	4Z→5Z	0.2549	0.1937	0.1689
1	5Z→6Z	0.2180	0.1622	0.1403
1	6Z→7Z	0.1635	0.1962	0.1535
2	3Z→4Z	0.2618	0.3199	0.2509
2	4Z→5Z	0.3248	0.2820	0.2240
2	5Z→6Z	0.2776	0.2083	0.1871
2	6Z→7Z	0.2269	0.1955	0.1201
3	4Z→5Z	0.2915	0.3192	0.2374
3	5Z→6Z	0.2842	0.2757	0.2472
3	6Z→7Z	0.2456	0.2243	0.1899
4	5Z→6Z	0.2994	0.3184	0.2363
4	6Z→7Z	0.2742	0.2718	0.2249
5	6Z→7Z	0.2910	0.3006	0.2350

Table S8. Deviation from perfect nesting, $\delta = 1-\Omega$, of cumulative levels $L_1 \oplus \cdots \oplus L_K$ built in neighbouring basis sets (hydrides, CCSD densities; mean over systems).

K	pair	standard	frozen-NMB	shell-nested
1	2Z→3Z	0.2081	0.1812	0.1448
1	3Z→4Z	0.1187	0.1286	0.0989
1	4Z→5Z	0.1121	0.0991	0.0566
1	5Z→6Z	0.0691	0.0836	0.0310
1	6Z→7Z	0.0778	0.1172	0.0660
2	3Z→4Z	0.1873	0.2318	0.1339
2	4Z→5Z	0.2004	0.1907	0.0856
2	5Z→6Z	0.1834	0.1833	0.0677
2	6Z→7Z	0.1612	0.1550	0.0780
3	4Z→5Z	0.2370	0.2941	0.1426
3	5Z→6Z	0.2062	0.2190	0.0950
3	6Z→7Z	0.1906	0.2031	0.0964
4	5Z→6Z	0.2535	0.2925	0.1543
4	6Z→7Z	0.2278	0.2560	0.0938
5	6Z→7Z	0.2411	0.2774	0.1495

Table S9. Deviation from perfect nesting, $\delta = 1-\Omega$, of cumulative levels $L_1 \oplus \cdots \oplus L_K$ built in neighbouring basis sets (rings, B3LYP densities; mean over systems).

K	pair	standard	frozen-NMB	shell-nested
1	2Z→3Z	0.3332	0.2650	0.2412
1	3Z→4Z	0.4010	0.2024	0.1239
1	4Z→5Z	0.3276	0.2563	0.1752
1	5Z→6Z	0.2538	0.2489	0.1809
1	6Z→7Z	0.2498	0.2838	0.1923
2	3Z→4Z	0.2860	0.3289	0.2195
2	4Z→5Z	0.2678	0.2844	0.1919
2	5Z→6Z	0.2596	0.2277	0.1740
2	6Z→7Z	0.2732	0.2966	0.2189
3	4Z→5Z	0.2958	0.3411	0.2172
3	5Z→6Z	0.2896	0.3183	0.1920
3	6Z→7Z	0.2750	0.2931	0.2226
4	5Z→6Z	0.3048	0.3172	0.2256
4	6Z→7Z	0.3059	0.3114	0.1883
5	6Z→7Z	0.2768	0.3006	0.2236

Table S10. Calibration anchors of the subspace-overlap measure Ω, Eq. (3) of the main text, over all systems and basis-set pairs.

Anchor	min Ω	max Ω
Occupied RHF space, basis a vs. b	0.997686	0.9999999
Random subspace of matching dimension	0.0309	0.0438

Table S11. Correlation energy recovered per hierarchy level (%full-parent value; shell-nested/FNO, both from the MP2 unrelaxed density, matched ranks, RI-MP2, cc-pV7Z parent).

System	L_1	L_2	L_3	L_4	L_5
BH_3	74.4/83.8	89.8/94.7	95.7/97.9	98.3/99.2	99.5/99.8
CH_4	72.4/84.6	88.7/95.1	95.0/98.2	98.2/99.4	99.4/99.8
NH_3	65.7/83.5	86.7/94.8	95.0/98.1	98.1/99.4	99.3/99.9
H_2O	65.1/80.0	85.7/94.1	94.5/97.8	98.0/99.3	99.3/99.9
HF	66.4/75.1	85.8/91.5	94.2/97.0	97.7/99.0	99.2/99.9
Ne	68.3/68.3	86.8/86.8	94.5/94.5	97.7/97.7	99.2/99.2

Table S12. Prediction of the full-parent correlation energy from three-level windows: absolute deviation (mE_h) of the fitted limit of Eq. (4) of the main text from the cc-pV7Z full-space value. "–" marks non-saturating windows.

System	window	shell-nested (MP2)	FNO (MP2)	shell-nested (RHF)
BH_3	L1–L3	1.780	1.245	3.232
	L2–L4	0.245	0.160	1.585
	L3–L5	0.372	0.175	1.223
CH_4	L1–L3	4.572	1.882	15.970
	L2–L4	1.667	0.137	13.888
	L3–L5	0.005	0.240	0.198
NH_3	L1–L3	2.307	1.999	1.426
	L2–L4	0.960	0.080	17.858
	L3–L5	0.067	0.351	4.967
H_2O	L1–L3	1.301	3.202	1.612
	L2–L4	0.384	0.230	2.382
	L3–L5	0.210	0.652	14.966
HF	L1–L3	1.891	2.671	–
	L2–L4	0.910	0.006	97.193
	L3–L5	0.905	1.215	33.068
Ne	L1–L3	1.599	1.599	135.577
	L2–L4	1.444	1.444	–
	L3–L5	1.520	1.520	–

Table S13. Formal limits from the deepest window (L_4–L_6) versus the external cc-pV5Z–cc-pV7Z limit $E_\infty^{\mathrm{cardinals}}$ (in E_h): absolute deviations (mE_h) for the shell-nested and FNO hierarchies built from the same MP2 density.

System	$E_\infty^{\mathrm{cardinals}}$	shell-nested	FNO
BH_3	-0.125182	0.108	0.394
CH_4	-0.218166	0.192	0.731
NH_3	-0.263319	0.417	1.143
H_2O	-0.298876	0.247	1.682
HF	-0.317649	0.421	2.200
Ne	-0.317548	0.724	0.724

Table S14. Model dependence of the window extrapolation of Sec. 4.5: median (over the five molecules) absolute deviation (mE_h) of the internal window limit from the external cc-pV5Z–cc-pV7Z limit, for six extrapolation models applied consistently to both. Superscripts count non-saturating windows.

Model	window	NAO	frozen-NMB	shell-nested	FNO
Feller e^{-bn}	L_2–L_4	23.45	45.55	1.38	1.17
	L_3–L_5	6.91	6.64	1.13	1.00
	L_4–L_6	0.70	0.71	0.31	1.40
Helgaker n^{-3}	L_2–L_4	13.05	15.12	4.29	2.71
	L_3–L_5	1.34	0.39	1.04	1.48
	L_4–L_6	2.77	2.85	0.39	1.60
free power $n^{-\alpha}$	L_2–L_4	21.49	24.66	8.21	0.84
	L_3–L_5	21.57	20.14	0.90	1.03
	L_4–L_6	1.25	1.22	0.62	2.32
$n^{-3}+n^{-5}$	L_2–L_4	2.94	1.55	0.32	1.23
	L_3–L_5	7.17	7.55	0.57	1.32
	L_4–L_6	0.78	0.53	0.27	2.34
shifted $(n+\delta)^{-3}$	L_2–L_4	11.77	12.08	2.24	0.28
	L_3–L_5	10.71	12.74	0.44	1.27
	L_4–L_6	1.29	1.31	0.46	1.97
root-exp. $e^{-\alpha\sqrt{x}}$	L_2–L_4	12.89	23.86	2.11	1.31
	L_3–L_5	4.67	4.54	1.30	0.88
	L_4–L_6	0.58	0.55	0.25	1.14

S4 Level extrapolation for the 32-molecule set in cc-pV5Z

Tables S15–S17 support the main-text subsection "Level extrapolation within a single basis set." For each of the 32 molecules, the RHF reference and the unrelaxed RI-MP2 density were obtained in cc-pV5Z; the shell-nested hierarchy and the FNO sequence were built from this density, and frozen-core RI-MP2 energies (AutoAux auxiliary basis, correlation-energy window as described in the section on the construction of level-truncated virtual spaces below) were evaluated in the level-truncated spaces L_2 and L_3 and in the FNO spaces of the same dimensions d_2 and d_3. Level L_4 is the complete cc-pV5Z virtual space of dimension d_4; its energy is shared by both sequences and coincides with the full-space cc-pV5Z energy to all reported digits. Table S15 lists the dimensions and energies together with the full-space cc-pVTZ and cc-pVQZ energies at the same geometries. Table S16 lists the three-point exponential limits of levels 2–4 (Eq. (4) of the main text, exact for three equally spaced points), the two-point n^{-3} estimate $E_{\mathrm{CBS}}^{(\mathrm{Q},5)}$, and the signed deviations; the last column applies the same three-point exponential fit to the full cc-pVTZ, cc-pVQZ, and cc-pV5Z energies. The requirement of decreasing increments, $|E_4 - E_3| < |E_3 - E_2|$, is met by both sequences for all 32 molecules. Against the n^{-3} estimate from cc-pVTZ and cc-pVQZ the mean absolute deviations are 3.2 (SNAO) and 9.5 mE_h (FNO), the SNAO limit being the closer one in 30 molecules; against the exponential fit to the three cardinals they are 4.9 and 7.5 mE_h, with the SNAO limit closer in 28. Table S17 gives the corresponding data for the window of levels 1–3, in which the FNO spaces are matched to the dimensions of L_1, L_2, and L_3; here the two limits are not distinguished (mean absolute deviations 10.3 and 11.6 mE_h, the SNAO limit closer in 19 molecules), and moving to the window of levels 2–4 reduces the SNAO deviation in 31 molecules and increases the FNO deviation in 27. The per-molecule data are provided as machine-readable tables (`paperA_L2L4_matched.tsv`, `paperA_L1L3_matched.tsv`, `paperA_5z_snao_levels.tsv`, `paperA_5z_fno_ranks.tsv`, `paperA_5z_full_TQ5.tsv`) with the generating scripts.

For the three rings (Table 2 of the main text, RI-SCS-MP2 in a cc-pV5Z parent), the same L_2–L_4 window gives shell-nested limits of -1.05179, -0.94504, and $-0.97356\,E_h$ and FNO limits of -1.02574, -0.91616, and $-0.94153\,E_h$ for benzene, pyrrole, and furan; the two-point cc-pV(Q,5)Z estimates from the full SCS-MP2 energies are -1.04908, -0.93800, and $-0.96546\,E_h$. In the window of levels 1–3 (dimensions 93/243/489, 77/202/407, and 72/188/377) the shell-nested limits deviate from these estimates by -1.7, -4.0, and $+2.8\,\mathrm{m}E_h$ and the FNO limits by $+19.7$, $+17.9$, and $+19.0\,\mathrm{m}E_h$.

Table S15. Correlation energies (frozen-core RI-MP2, E_h) in the level-truncated virtual spaces of the cc-pV5Z parent for the 32-molecule set: active virtual dimensions of levels L_2, L_3, and L_4 (the full space), energies in the shell-nested (SNAO) levels and in the rank-matched FNO spaces, and the full-space cc-pVTZ, cc-pVQZ, and cc-pV5Z energies.

molecule	d_2	d_3	d_4	$E^{\mathrm{SNAO}}(L_2)$	$E^{\mathrm{SNAO}}(L_3)$	$E^{\mathrm{FNO}}(d_2)$	$E^{\mathrm{FNO}}(d_3)$	$E(\mathrm{T})$	$E(\mathrm{Q})$	$E(5) = E(L_4)$
F_2	51	101	173	-0.525472	-0.570247	-0.536065	-0.573866	-0.521109	-0.568154	-0.588138
N_2	53	103	175	-0.376168	-0.398220	-0.385039	-0.402262	-0.372717	-0.397782	-0.408139
CO	53	103	175	-0.356424	-0.380865	-0.368437	-0.385902	-0.355458	-0.381304	-0.391984
HCN	67	133	230	-0.344333	-0.366445	-0.359126	-0.372361	-0.344703	-0.366963	-0.375871
H_2CO	80	162	284	-0.392866	-0.421255	-0.415490	-0.431996	-0.395500	-0.424007	-0.435509
CH_3OH	107	221	393	-0.423931	-0.456890	-0.452782	-0.469200	-0.428614	-0.459906	-0.472278
NH_2OH	93	191	338	-0.458404	-0.498934	-0.493914	-0.513302	-0.467594	-0.502874	-0.517163
H_2O_2	79	161	283	-0.492731	-0.533411	-0.523182	-0.547929	-0.497616	-0.537001	-0.553295
N_2H_4	107	221	393	-0.426998	-0.466683	-0.463643	-0.480124	-0.440298	-0.471299	-0.483526
CH_3NH_2	121	251	448	-0.395270	-0.427803	-0.424509	-0.438646	-0.404777	-0.431514	-0.441742
CH_3F	93	191	338	-0.433957	-0.469460	-0.462449	-0.481378	-0.434601	-0.470280	-0.484778
C_2H_2	81	163	285	-0.310132	-0.328317	-0.323392	-0.333816	-0.309398	-0.328739	-0.336214
C_2H_4	108	222	394	-0.330008	-0.353914	-0.349911	-0.361191	-0.335371	-0.356062	-0.363841
C_2H_6	135	281	503	-0.364739	-0.390127	-0.385601	-0.397616	-0.370141	-0.392401	-0.400632
propyne	135	274	482	-0.477118	-0.509112	-0.503032	-0.519413	-0.481891	-0.511848	-0.523155
allene	135	274	482	-0.472161	-0.504626	-0.497175	-0.513943	-0.476784	-0.506656	-0.517860
cyclopropane	162	333	591	-0.506148	-0.543024	-0.538849	-0.556182	-0.516856	-0.548231	-0.560165
CO_2	79	154	262	-0.599361	-0.642043	-0.623795	-0.655133	-0.601795	-0.647135	-0.665766
CH_2CO	107	214	372	-0.533253	-0.573228	-0.564242	-0.586646	-0.539427	-0.576948	-0.591846
CH_3CHO	134	273	481	-0.560974	-0.603530	-0.596370	-0.618100	-0.567769	-0.607037	-0.622558
oxirane	134	273	481	-0.566794	-0.609219	-0.605029	-0.626938	-0.575732	-0.615509	-0.631403
C_2H_5OH	161	332	590	-0.593512	-0.639933	-0.633568	-0.655908	-0.602332	-0.644104	-0.660371
$(CH_3)_2O$	161	332	590	-0.585126	-0.635010	-0.629560	-0.651947	-0.598927	-0.640265	-0.656418
HCOOH	106	213	371	-0.624080	-0.673775	-0.661268	-0.691061	-0.630696	-0.678459	-0.697912
N_2O	79	154	262	-0.639558	-0.682040	-0.662323	-0.693789	-0.642902	-0.686876	-0.704766
O_3	78	153	261	-0.772282	-0.827562	-0.794880	-0.836941	-0.774853	-0.829105	-0.852054
OF_2	77	152	260	-0.758772	-0.825708	-0.781765	-0.834068	-0.760291	-0.825576	-0.853421
CH_2F_2	105	212	370	-0.673524	-0.732496	-0.709226	-0.748033	-0.674099	-0.733097	-0.757536
HN_3	93	184	317	-0.606542	-0.651381	-0.634863	-0.661490	-0.613014	-0.653220	-0.669283
HNO_2	92	183	316	-0.679849	-0.732154	-0.710187	-0.745483	-0.684064	-0.734875	-0.755994
FNO	78	153	261	-0.700911	-0.756411	-0.723460	-0.765253	-0.701146	-0.756732	-0.780211
HNCO	93	184	317	-0.572472	-0.615718	-0.602789	-0.629289	-0.578234	-0.619735	-0.636509

Table S16. Three-point exponential limits (levels 2–4) of the SNAO and rank-matched FNO sequences for the 32-molecule set, the two-point n^{-3} estimate from the full cc-pVQZ and cc-pV5Z energies, and the signed deviations $E_\infty - E_{\mathrm{CBS}}^{(\mathrm{Q},5)}$ (mE_h). The last column gives the deviation of the same three-point exponential fit applied to the full cc-pVTZ, cc-pVQZ, and cc-pV5Z energies.

molecule	E_∞^{SNAO}	E_∞^{FNO}	$E_{\mathrm{CBS}}^{(\mathrm{Q},5)}$	Δ^{SNAO}	Δ^{FNO}	$\Delta^{(\mathrm{T,Q},5)}$
F_2	-0.600043	-0.596794	-0.609104	+9.06	+12.31	+6.21
N_2	-0.416248	-0.411184	-0.419005	+2.76	+7.82	+3.57
CO	-0.401263	-0.395234	-0.403189	+1.93	+7.96	+3.68
HCN	-0.382875	-0.377138	-0.385218	+2.34	+8.08	+3.40
H_2CO	-0.449880	-0.436458	-0.447576	-2.30	+11.12	+4.29
CH_3OH	-0.485756	-0.472989	-0.485259	-0.50	+12.27	+4.89
NH_2OH	-0.532064	-0.518123	-0.532156	+0.09	+14.03	+5.26
H_2O_2	-0.572308	-0.554781	-0.570390	-1.92	+15.61	+5.60
N_2H_4	-0.495946	-0.484411	-0.496354	+0.41	+11.94	+4.87
CH_3NH_2	-0.452190	-0.442610	-0.452473	+0.28	+9.86	+4.39
CH_3F	-0.496403	-0.485522	-0.499989	+3.59	+14.47	+5.29
C_2H_2	-0.342275	-0.336929	-0.344055	+1.78	+7.13	+3.13
C_2H_4	-0.370892	-0.364655	-0.372002	+1.11	+7.35	+3.48
C_2H_6	-0.408047	-0.401644	-0.409268	+1.22	+7.62	+3.81
propyne	-0.534142	-0.524263	-0.535019	+0.88	+10.76	+5.01
allene	-0.526969	-0.519055	-0.529616	+2.65	+10.56	+5.03
cyclopropane	-0.575055	-0.561354	-0.572687	-2.37	+11.33	+5.19
CO_2	-0.695447	-0.671226	-0.685313	-10.13	+14.09	+6.55
CH_2CO	-0.608076	-0.593417	-0.607476	-0.60	+14.06	+5.82
CH_3CHO	-0.637945	-0.623709	-0.638842	+0.90	+15.13	+6.14
oxirane	-0.655717	-0.632545	-0.648078	-7.64	+15.53	+6.10
C_2H_5OH	-0.676447	-0.661485	-0.677437	+0.99	+15.95	+6.69
$(CH_3)_2O$	-0.672513	-0.657534	-0.673366	+0.85	+15.83	+6.59
HCOOH	-0.720709	-0.699958	-0.718323	-2.39	+18.36	+7.04
N_2O	-0.730909	-0.710646	-0.723535	-7.37	+12.89	+6.50
O_3	-0.871537	-0.860531	-0.876132	+4.60	+15.60	+7.25
OF_2	-0.873002	-0.864787	-0.882636	+9.63	+17.85	+8.50
CH_2F_2	-0.776016	-0.760618	-0.783178	+7.16	+22.56	+8.36
HN_3	-0.681182	-0.672508	-0.686136	+4.95	+13.63	+6.17
HNO_2	-0.775961	-0.760452	-0.778153	+2.19	+17.70	+7.14
FNO	-0.798081	-0.788550	-0.804846	+6.77	+16.30	+7.46
HNCO	-0.655758	-0.639213	-0.654108	-1.65	+14.89	+6.22
mean absolute deviation				3.22	13.14	5.61
median absolute deviation				2.25	13.83	5.71
maximum absolute deviation				10.13	22.56	8.50

Table S17. Window of levels 1–3 for the 32-molecule set: active virtual dimension d_1 of level L_1, correlation energies in L_1 (SNAO) and in the FNO space of the same dimension (E_h), the three-point exponential limits of levels 1–3, and their signed deviations from $E_{\mathrm{CBS}}^{(\mathrm{Q},5)}$ (mE_h). Levels 2 and 3 as in Table S15.

molecule	d_1	$E^{\mathrm{SNAO}}(L_1)$	$E^{\mathrm{FNO}}(d_1)$	$E_{\infty}^{\mathrm{SNAO}}$	$E_{\infty}^{\mathrm{FNO}}$	Δ^{SNAO}	Δ^{FNO}
F_2	19	-0.410042	-0.435124	-0.598621	-0.596497	+10.48	+12.61
N_2	21	-0.309245	-0.333807	-0.409057	-0.410984	+9.95	+8.02
CO	21	-0.285646	-0.317394	-0.393758	-0.394987	+9.43	+8.20
HCN	26	-0.286739	-0.315356	-0.380226	-0.378098	+4.99	+7.12
H_2CO	30	-0.309496	-0.357201	-0.435915	-0.438515	+11.66	+9.06
CH_3OH	39	-0.331752	-0.392250	-0.475233	-0.475310	+10.03	+9.95
NH_2OH	34	-0.357757	-0.420599	-0.526260	-0.520273	+5.90	+11.88
H_2O_2	29	-0.381288	-0.439583	-0.556796	-0.558334	+13.59	+12.06
N_2H_4	39	-0.336867	-0.402453	-0.497902	-0.486200	-1.55	+10.15
CH_3NH_2	44	-0.315705	-0.373768	-0.450308	-0.444106	+2.17	+8.37
CH_3F	34	-0.334307	-0.389265	-0.489109	-0.487983	+10.88	+12.01
C_2H_2	31	-0.260140	-0.286612	-0.338714	-0.337939	+5.34	+6.12
C_2H_4	40	-0.271411	-0.309324	-0.370386	-0.365533	+1.62	+6.47
C_2H_6	49	-0.296156	-0.342328	-0.405048	-0.402233	+4.22	+7.04
propyne	51	-0.397172	-0.444541	-0.530458	-0.525785	+4.56	+9.23
allene	51	-0.391247	-0.438215	-0.526380	-0.520607	+3.24	+9.01
cyclopropane	60	-0.406436	-0.475487	-0.564664	-0.562710	+8.02	+9.98
CO_2	31	-0.474545	-0.531843	-0.664224	-0.671335	+21.09	+13.98
CH_2CO	41	-0.430963	-0.486773	-0.598871	-0.595762	+8.60	+11.71
CH_3CHO	50	-0.444175	-0.516727	-0.627925	-0.626253	+10.92	+12.59
oxirane	50	-0.447523	-0.524735	-0.632641	-0.635160	+15.44	+12.92
C_2H_5OH	59	-0.469408	-0.552745	-0.667672	-0.664442	+9.77	+13.00
$(CH_3)_2O$	59	-0.460198	-0.548638	-0.668169	-0.660509	+5.20	+12.86
HCOOH	40	-0.484888	-0.561739	-0.701369	-0.703790	+16.95	+14.53
N_2O	31	-0.515861	-0.570896	-0.704260	-0.710302	+19.28	+13.23
O_3	30	-0.607853	-0.677424	-0.855560	-0.860405	+20.57	+15.73
OF_2	29	-0.585179	-0.640468	-0.867717	-0.864808	+14.92	+17.83
CH_2F_2	39	-0.508413	-0.586108	-0.765260	-0.765896	+17.92	+17.28
HN_3	36	-0.498652	-0.550865	-0.683268	-0.673848	+2.87	+12.29
HNO_2	35	-0.526853	-0.601746	-0.759324	-0.762515	+18.83	+15.64
FNO	30	-0.543911	-0.607892	-0.786759	-0.788927	+18.09	+15.92
HNCO	36	-0.461259	-0.517040	-0.643235	-0.641142	+10.87	+12.97
mean absolute deviation						10.28	11.55
median absolute deviation						9.99	12.03

S5 Matrix formulation of the shell-nested construction

This section restates the shell-nested construction of the main text in explicit matrix form; all conventions follow the main text ($\mathbf{S}$ = AO overlap, $\mathbf{D}$ = spinless one-electron density, $\mathbf{M} = \mathbf{SDS}$). After cycle k the frozen space is held as the $\mathbf{S}$-orthonormal column block $\mathbf{\Phi}_k = [\,\mathbf{C}_{\mathrm{NMB}}\,|\,\mathbf{C}_{L_1}\,|\cdots|\,\mathbf{C}_{L_{k-1}}\,]$ (cycle 1 itself is the frozen-NMB construction), and the working complement is reached through the projector

$$\mathbf{Q}_k \;=\; \mathbf{1} \;-\; \mathbf{\Phi}_k\mathbf{\Phi}_k^{\mathsf{T}}\mathbf{S}. \tag{S4}$$

Cycle $k{+}1$ then performs, for every channel (A, l) admitted by the eligibility rule of Eq. (2) of the main text, the following steps.

(a) Residual channel space. Let $\mathbf{B}^{(m)}$ collect the AO coefficient columns of the radial basis functions of channel (A, l) at magnetic component m. The residual channel basis is $\mathbf{R}^{(m)} = \mathbf{Q}_k\mathbf{B}^{(m)}$, orthonormalized canonically through the m-averaged metric

$$\boldsymbol{\sigma} = \frac{1}{2l{+}1}\sum_m \mathbf{R}^{(m)\mathsf{T}}\mathbf{S}\,\mathbf{R}^{(m)} = \mathbf{U}\,\mathrm{diag}(s)\,\mathbf{U}^{\mathsf{T}}, \qquad \mathbf{X}^{(m)} = \mathbf{R}^{(m)}\,\mathbf{U}_>\,\mathrm{diag}(s_>^{-1/2}), \tag{S5}$$

where $\mathbf{U}_>$ retains eigenvalues above the relative linear-dependence threshold (directions already represented in $\mathbf{\Phi}_k$ appear here as numerical nulls and are removed). The single radial rotation is applied identically to all $2l{+}1$ components, which keeps the construction covariant under molecular rotations.

(b) Natural radial function. The m-averaged occupation matrix

$$\bar{\mathbf{M}}_{uv} = \frac{1}{2l{+}1}\sum_m \mathbf{x}_u^{(m)\mathsf{T}}\,\mathbf{M}\,\mathbf{x}_v^{(m)} \tag{S6}$$

is diagonalized, and the eigenvector $\mathbf{q}_1$ with the largest eigenvalue—the maximizer of the Rayleigh quotient of Eq. (1) of the main text over the residual channel space—defines the selected vector $\mathbf{v}^{(m)} = \mathbf{X}^{(m)}\mathbf{q}_1$, replicated over all m.

(c) Layer assembly and freezing. The selected vectors of all eligible channels are collected into $\mathbf{W} = [\ldots\ \mathbf{v}\ \ldots]$, re-projected once, $\mathbf{W} \leftarrow \mathbf{Q}_k\mathbf{W}$, and orthonormalized by occupation-weighted symmetric orthogonalization with weights $w_i = \max(n_i, 10^{-6})$,

$$\mathbf{W}' = \mathbf{W}\,\mathbf{\Omega}\,\big(\mathbf{\Omega}\,\mathbf{W}^{\mathsf{T}}\mathbf{S}\,\mathbf{W}\,\mathbf{\Omega}\big)^{-1/2}, \qquad \mathbf{\Omega} = \mathrm{diag}(w_i), \tag{S7}$$

followed by a final symmetric (Löwdin) orthonormalization $\mathbf{W}'' = \mathbf{W}'\big(\mathbf{W}'^{\mathsf{T}}\mathbf{S}\,\mathbf{W}'\big)^{-1/2}$ to machine precision. If the weighted overlap in Eq. (S7) is numerically singular (a rare cross-atom degeneracy among diffuse layer members), the lowest-occupation channel group of the layer is dropped, that channel is retired, and the orthonormalization is repeated. The layer is then frozen, $\mathbf{\Phi}_{k+1} = [\,\mathbf{\Phi}_k\,|\,\mathbf{W}''\,]$. Both Eq. (S7) and the final orthonormalization form linear combinations inside $\mathrm{span}(\mathbf{W})$ only, so previously frozen columns are never rotated and the level spaces are nested by construction. The recursion terminates when every radial direction has been selected; orbital labels follow $n(l) = l + 1 + u$ as in the main text.

S6 Construction of the level-truncated virtual spaces

The correlated calculations discussed under "Compression and extrapolation are different tasks are performed in virtual spaces truncated to a chosen hierarchy level, with the RHF reference

determinant left exactly unchanged. The transformation from the canonical orbitals and a NAO hierarchy to such a calculation proceeds in five steps; $\mathbf{S}$ denotes the AO overlap matrix, $\mathbf{C}_{\mathrm{occ}}$ the canonical occupied orbitals with energies ε_i, and $\mathbf{F}$ the converged Fock matrix (reconstructed, where needed, as $\mathbf{F} = \mathbf{S}\,\mathbf{C}\,\mathrm{diag}(\varepsilon)\,\mathbf{C}^{\mathsf{T}}\mathbf{S}$ from the full canonical set).

1. *Selection.* All NAO columns with level index up to the target K are gathered (the NMB together with levels $1, \ldots, K$); for the standard and frozen-NMB constructions the columns are selected by the principal-label rule described in Computational Methods, and for the FNO sequence the leading natural orbitals of the MP2 unrelaxed density are taken at the matching dimension.

2. *Projection.* The selected columns are projected onto the orthogonal complement of the occupied space, $\mathbf{A} = \big(\mathbf{1} - \mathbf{C}_{\mathrm{occ}}\mathbf{C}_{\mathrm{occ}}^{\mathsf{T}}\mathbf{S}\big)\mathbf{C}_{\mathrm{sel}}$, so that the occupied orbitals—and with them the reference determinant and its energy—remain exactly those of the canonical calculation.

3. *Dimension-controlled orthonormalization.* The projected set is orthonormalized canonically in the $\mathbf{S}$ metric. Exactly n_{occ} null directions, corresponding to the occupied component removed in step 2, are discarded by count, together with any remaining directions below the relative linear-dependence threshold. The result is an orthonormal active virtual set of dimension $d_K = n_{\mathrm{sel}} - n_{\mathrm{occ}}$.

4. *Semicanonicalization.* The Fock matrix is diagonalized within the active virtual set, yielding active virtual orbitals with well-defined orbital energies; this step only rotates the active set and leaves its span unchanged.

5. *Exclusion of the complement.* The orthonormal complement of the occupied and active spaces is retained to complete the orbital set, with its orbital energies set to a $10^4\,E_h$ placeholder; the correlation energy window $(-3, 10^3)\,E_h$ of the subsequent calculation then excludes these orbitals, together with the frozen core, from the correlation treatment.

The assembled orbital set is written as a standard orbital file and the RI-MP2 calculation is restarted from it without SCF iterations. For the shell-nested hierarchy steps 1–5 are performed natively by RUNSNVO; for the label-selected and FNO sets an equivalent external implementation of steps 2–5 was used and validated against the native path: for a shell-nested test level the two routes agree in the correlated energy to $8 \times 10^{-9}\,E_h$. The level-6 calculation of every hierarchy recovers the full-space correlation energy to all reported digits (main-text Computational Methods), which verifies the completeness of the assembled sets.

S7 Precision limits of interchange formats for deep Rydberg orbitals

The ring benchmark uses the Gaussian formatted-checkpoint (fchk) format to carry the AO→NAO transformation, which stores real numbers with nine significant digits. For the shell-nested and frozen-NMB constructions this precision is ample: the largest deviation from $\mathbf{S}$-orthonormality of any transported NAO set, measured as $\max|\mathbf{C}^{\mathsf{T}}\mathbf{S}\mathbf{C} - \mathbf{I}|$ against analytically recomputed overlap integrals, is 1.4×10^{-4} across all rings and cardinals, dominated by benign rounding. For the standard full-space construction, the deepest, nearly unoccupied Rydberg NAOs in the larger basis sets can have large, mutually cancelling AO coefficients.[1] Nine significant digits may then be insufficient to preserve those cancellations: individual columns lose their normalization by as much as 0.37 (the nitrogen $6s$ NAO of pyrrole at cc-pV5Z), with

the error increasing along that channel ($3s$: 4×10^{-3}, $4s$: 4×10^{-2}, $5s$: 8×10^{-2}, $6s$: 0.37). The affected rows are identified in the machine-readable tables through the `gauge_err` column. All subspace comparisons in the main text use analytically validated shell-nested columns as the reference representation. This is a file-precision consequence of the large coefficients described in the frozen-NMB study, not a change in the underlying real-space orbital; it is not observed for either frozen construction.

S8 Terminology and program options

Table S18. Mapping between the terminology of this paper and the RUNSNVO command-line options used to generate the data.

This paper	RUNSNVO
standard construction	`-nao-algorithm nao`
frozen-NMB construction	`-nao-algorithm fnmb`
shell-nested construction (SNAO)	`-nao-algorithm snao`
shell-resolved population table	`-table shell`
per-NAO population table	`-table ao`
NAO set, ORCA-convention export	`-export-json ao`
NAO set, fchk export	`-export-fchk ao`
NAO cube files	`-export-cube ao[...]`
level-truncated virtual spaces L_k	`-export-json snvo -snvo-rhf`
FNO spaces at matched dimension	`-export-json fno -fno-vo`

The program also accepts the earlier option names `rigorous`, `frozen-nmb`, and `cc-layers` as aliases of `nao`, `fnmb`, and `snao`.

S9 Geometries

All hydride calculations use fixed experimental geometries taken from the NIST Computational Chemistry Comparison and Benchmark Database (CCCBDB, `http://cccbdb.nist.gov/`), in Å:

```
B    0.0000   0.0000   0.0000
H    0.0000   1.1900   0.0000
H    1.0306  -0.5950   0.0000
H   -1.0306  -0.5950   0.0000

C    0.0000   0.0000   0.0000
H    0.6276   0.6276   0.6276
H    0.6276  -0.6276  -0.6276
H   -0.6276   0.6276  -0.6276
H   -0.6276  -0.6276   0.6276

N    0.0000   0.0000   0.0000
H    0.0000  -0.9377  -0.3816
H    0.8121   0.4689  -0.3816
H   -0.8121   0.4689  -0.3816
```

[1] See the preceding frozen-NMB study (main text, ref. 4) for the origin of these large AO coefficients near the CBS limit.

```
O    0.0000   0.0000   0.1173
H    0.0000   0.7572  -0.4692
H    0.0000  -0.7572  -0.4692

F    0.0000   0.0000   0.0000
H    0.0000   0.0000   0.9168
```

(The neon atom needs no geometry.)

The ring geometries are the B3LYP-D3BJ/cc-pV5Z equilibrium structures of the preceding paper (its Supporting Information); all basis-set sequences share one fixed geometry per molecule.

The 32 molecules used for the single-basis extrapolation benchmark were optimized at the r^{2}SCAN-3c level with ORCA 6.1.1 (tight convergence, no symmetry constraints) starting from PubChem3D structures (F_2 and allene from experimental parameters); harmonic frequencies confirm all structures as minima. Coordinates in Å:

```
F2
F       0.000000     0.000000     0.006005
F       0.000000     0.000000     1.405995

N2
N      -0.547030     0.000000     0.000000
N       0.547030     0.000000     0.000000

CO
O       0.565226     0.000000     0.000000
C      -0.565226     0.000000     0.000000

HCN
N      -0.575141    -0.000000     0.000000
C       0.575495     0.000000    -0.000000
H       1.644646    -0.000000     0.000000

H2CO
O       0.597146    -0.000014    -0.000007
C      -0.606015     0.000050    -0.000000
H      -1.195558     0.245646    -0.911165
H      -1.195573    -0.245482     0.911172

CH3OH
O       0.720998     0.001565     0.000841
C      -0.704940    -0.016014    -0.005328
H      -1.114814    -0.767558     0.685302
H      -1.114519    -0.193754    -1.010558
H      -1.033255     0.971367     0.328684
H       1.030631    -0.861106    -0.291840

NH2OH
O       0.735972     0.004443    -0.024720
```

```
N      -0.715978    -0.002074     0.011842
H      -0.940495    -0.900331     0.437518
H      -0.940520     0.693082     0.722328
H       0.917621     0.171079    -0.956867

H2O2
O       0.729157    -0.061145     0.006614
O      -0.729161    -0.061505     0.000564
H       0.908517    -0.643109    -0.747004
H      -0.908513    -0.551741     0.816826

N2H4
N       0.719537    -0.027646    -0.076057
N      -0.719505    -0.080528     0.008033
H       1.083281    -0.328270     0.821386
H       1.081690    -0.670567    -0.777119
H      -1.081686    -0.990884    -0.267795
H      -1.083316     0.598195    -0.651548

CH3NH2
N      -0.741701    -0.000697    -0.006792
C       0.728027     0.001763     0.019983
H       1.180854     0.091790     1.021721
H       1.099274     0.830916    -0.591869
H       1.099279    -0.923131    -0.434351
H      -1.092354     0.849756     0.420280
H      -1.092280    -0.760997     0.565629

CH3F
F       0.709082    -0.000012     0.000004
C      -0.683587    -0.000025    -0.000022
H      -1.037808     0.465091     0.926012
H      -1.037808     0.569383    -0.865838
H      -1.037779    -1.034536    -0.060256

C2H2
C      -0.600222     0.000000    -0.000003
C       0.600222    -0.000000     0.000003
H      -1.663924    -0.000000    -0.000099
H       1.663924    -0.000000     0.000099

C2H4
C      -0.663399     0.000006    -0.000000
C       0.663432    -0.000006     0.000000
H      -1.232980    -0.922705     0.070320
H      -1.232963     0.922728    -0.070322
H       1.233013     0.922706    -0.070320
H       1.232997    -0.922728     0.070322
```

```
C2H6
C      -0.763529     0.000024    -0.000008
C       0.763529    -0.000024     0.000008
H      -1.161339     0.655808     0.781172
H      -1.161330     0.348632    -0.958526
H      -1.161364    -1.004394     0.177355
H       1.161330    -0.348635     0.958525
H       1.161364     1.004394    -0.177353
H       1.161339    -0.655806    -0.781174

C3H4 (propyne)
C      -1.370101     0.000120    -0.000042
C       0.079905     0.000085    -0.000086
C       1.283661     0.000144    -0.000110
H      -1.759992     0.065983     1.020983
H      -1.760074     0.851414    -0.567586
H      -1.760080    -0.917032    -0.453512
H       2.346480     0.000086    -0.000048

C3H4 (allene)
C      -0.000000     0.000000     0.000000
C      -0.000000     0.000001     1.301333
C      -0.000000     0.000000    -1.301333
H       0.930455    -0.000000     1.863282
H      -0.930455    -0.000000     1.863282
H      -0.000000     0.930455    -1.863282
H       0.000000    -0.930455    -1.863282

C3H6 (cyclopropane)
C       0.319155     0.807442     0.000014
C      -0.858868    -0.127309    -0.000018
C       0.539705    -0.680134     0.000004
H       0.534790     1.353119    -0.913074
H       0.534744     1.353100     0.913126
H      -1.439236    -0.213408     0.913098
H      -1.439217    -0.213400    -0.913146
H       0.904426    -1.139685    -0.913125
H       0.904401    -1.139724     0.913122

CO2
O      -1.163426    -0.000000    -0.000000
O       1.163426    -0.000000    -0.000000
C      -0.000000     0.000000     0.000000

CH2CO
O       1.205791     0.000042     0.000113
C      -1.265808     0.000047    -0.000364
C       0.042324     0.000045    -0.000103
H      -1.799470     0.809789     0.478643
H      -1.799238    -0.809722    -0.479588
```

```
CH3CHO
O      1.152938    0.223440    0.000823
C     -1.249038    0.195125    0.000028
C      0.124216   -0.409608   -0.000179
H     -1.803016   -0.159678    0.878152
H     -1.196905    1.285414    0.001134
H     -1.802520   -0.157929   -0.879112
H      0.148926   -1.526064   -0.001346

C2H4O (oxirane)
O     -0.000590    0.791739   -0.000043
C      0.732422   -0.437767    0.000004
C     -0.731714   -0.438891   -0.000000
H      1.268492   -0.657400    0.923748
H      1.268502   -0.657399   -0.923732
H     -1.267451   -0.659409   -0.923727
H     -1.267461   -0.659274    0.923751

C2H5OH
O     -1.218900    0.277570   -0.004456
C     -0.046978   -0.541713    0.017105
C      1.237193    0.271949    0.000978
H     -0.061594   -1.218801    0.886748
H     -0.114585   -1.164483   -0.881315
H      2.112217   -0.387152   -0.030501
H      1.264424    0.929472   -0.873307
H      1.321483    0.893177    0.900760
H     -1.204159    0.834482    0.781687

(CH3)2O
O      0.000004    0.533320   -0.000031
C      1.171171   -0.262030    0.000004
C     -1.171150   -0.262051   -0.000029
H      1.223393   -0.904514    0.894530
H      2.026608    0.418244   -0.000000
H      1.223418   -0.904558   -0.894488
H     -1.223387   -0.904534    0.894497
H     -1.223358   -0.904582   -0.894522
H     -2.026599    0.418207   -0.000061

HCOOH
O     -1.183222    0.174298    0.000049
O      1.081470    0.225758   -0.000023
C      0.045622   -0.383174    0.000058
H     -0.049007   -1.480936    0.000158
H     -1.060262    1.138655   -0.000042

N2O
O      1.163772    0.000000   -0.000000
```

```
N       -0.018008    -0.000000     0.000000
N       -1.145764     0.000000    -0.000000

O3
O       -0.020209    -0.432108     0.000000
O        1.093648     0.165218     0.000000
O       -1.073539     0.266889     0.000000

OF2
F       -1.113147     0.287941     0.000000
F        1.113147     0.287941     0.000000
O       -0.000000    -0.575881     0.000000

CH2F2
F       -1.108998    -0.259247    -0.000000
F        1.108998    -0.259247    -0.000000
C        0.000000     0.539176     0.000000
H        0.000000     1.140609     0.917561
H       -0.000000     1.140609    -0.917561

HN3
N        0.040878    -0.044749     0.049643
N       -1.190623     0.000052    -0.000055
N        1.167019     0.030165    -0.033465
H       -1.601274    -0.624568     0.692977

HNO2
O       -1.144950     0.260262     0.000003
O        1.019490     0.227628    -0.000003
N        0.103152    -0.490199     0.000003
H       -1.809593    -0.447591    -0.000003

FNO
F        1.186636     0.256741     0.000000
O       -1.023750     0.237553     0.000000
N       -0.162886    -0.494294     0.000000

HNCO
O       -1.224720    -0.088742     0.044807
N        1.145828     0.064872    -0.032832
C       -0.068929     0.059177    -0.029972
H        1.697221     0.816493    -0.412704
```
